# Macroalgal–Coral Interactions in New Caledonia South West Lagoon: Diversity, Abundance, and Spatial Patterns

**DOI:** 10.3390/biology14101419

**Published:** 2025-10-15

**Authors:** Christophe Vieira, Christophe Peignon, Olivier De Clerck, Claude Payri

**Affiliations:** 1 Research Institute for Basic Sciences, Jeju National University, Jeju 63243, Republic of Korea; 2 UMR ENTROPIE, (IRD, UR, UNC, CNRS, Ifremer) B.P. A5, 98848 Nouméa Cedex, New Caledonia; christophe.peignon@ird.fr (C.P.); claude.payri@ird.fr (C.P.); 3 Phycology Research Group and Center for Molecular Phylogenetics and Evolution, Ghent University, Krijgslaan 281 (S8), B-9000 Gent, Belgium; olivier.declerck@ugent.be

**Keywords:** seaweeds, macoralgae, coral reefs, biotic interactions, competition, South Pacific, healthy reefs, interaction typology, Coralgal Biotic Interaction Compass (CBIC)

## Abstract

**Simple Summary:**

Coral reefs host both corals and macroalgae (seaweeds) that often live in close contact. While these interactions are well studied on degraded reefs, much less is known about how they occur in healthy reef systems. We surveyed macroalgal–coral interactions (MCI) across 26 habitats in the South West Lagoon of New Caledonia and found that these interactions are common and varied. On average, they covered 16% of the reef surface, involving 43 combinations of coral and macroalgal genera. The most frequent interactions involved *Lobophora*, *Hypnea* and *Halimeda* macroalgae with *Acropora*, *Montipora*, *Seriatopora* and *Porites* corals. Some interactions were far more common than others—for example, *Lobophora–Acropora* represented almost a third of all interactions. Their distribution also depended on habitat type, showing that these interactions are not random but shaped by the local environment and the identity of the taxon involved. We also describe six main forms of association between macroalgae and corals and introduce a new framework, the Coralgal Biotic Interaction Compass, to guide future studies. These findings show that MCI are a normal and structured feature of undisturbed reefs, providing essential baseline data for understanding coral reef ecology and resilience.

**Abstract:**

Macroalgal–coral interactions (MCI) are an integral yet understudied component of coral reef ecology, particularly in healthy systems where they may represent stable coexistence rather than competition. This study provides the first comprehensive assessment of MCI diversity, abundance, and spatial patterns in the South West Lagoon of New Caledonia (SWLNC). Across 26 coral-dominated habitats, MCI accounted for an average of 16.4% of the benthic cover, with local values reaching 70% in high-interaction areas. A total of 43 unique macroalgal–coral genus pairings were documented, involving 16 macroalgal and 10 coral genera. *Lobophora* (47%), *Halimeda* (20%), and *Hypnea* (9%) were the dominant macroalgae, while *Acropora* (61%), *Montipora* (19%), *Seriatopora* (13%), and *Porites* (5%) were the most frequent coral interactants. The most abundant specific interactions were *Lobophora–Acropora* (29%), *Hypnea–Acropora* (15%), *Halimeda–Montipora* (10%), *Lobophora–Seriatopora* (10%), and *Halimeda–Acropora* (10%). MCI abundance varied markedly among habitat levels, differing across reef types, zonation, and benthic cover. Six recurrent typologies of physical association were identified, and the Coralgal Biotic Interaction Compass (CBIC) is introduced as a conceptual framework to distinguish the nature of macroalgal-coral associations. Overall, the study demonstrates that MCI in the SWLNC are diverse, structured, and non-random, shaped by both interactant identity and habitat filtering rather than ubiquity, providing a robust ecological baseline for future analyses of macroalgal-coral dynamics in Indo-Pacific reef systems.

## 1. Introduction

Corals and macroalgae, as integral components of benthic reef ecosystems, significantly influence the intricate diversity found within tropical coral reefs [1,2,3]. Macroalgae offer a range of critical services to the normal functioning of coral reef systems. These include serving as primary producers, contributing significantly to reef productivity, providing critical habitat and refuge for various invertebrates and juvenile fish, facilitating nutrient cycling, and stabilizing sediments [4,5,6,7]. They also serve as a crucial food source for herbivorous fishes and invertebrates, which are key to maintaining ecological balance on reefs [8,9].

The prevailing notion suggests that in a pristine state of tropical coral reefs, the presence of macroalgae was minimal, often estimated at less than 2% based on Caribbean surveys [10,11]. Additionally, it was believed that in these “normal”—pristine—reefs, spatial segregation between coral and macroalgal communities was established, primarily driven by the competitive advantages of corals through herbivory and defense mechanisms [12,13]. Disturbances, whether natural or anthropogenic, were thought to disrupt this equilibrium, allowing macroalgae or other reef organisms to exploit altered conditions and potentially shift the ecosystem towards a macroalgal-dominated state [14]. These observed shifts underscored the significance of competitive interactions between corals and macroalgae, leading to a perception of macroalgae as threats to coral resilience, e.g., [13,15].

However, mounting evidence from remote quasi-pristine Pacific reefs has defied conventional notions, revealing macroalgal coverage ranging from 10% to 30% [16,17]. This apparent discrepancy in macroalgal cover between Caribbean and Indo-Pacific reefs is largely attributed to fundamental differences in their ecological histories and prevailing anthropogenic pressures. Caribbean reefs, for instance, have been profoundly impacted by widespread disturbances such as the 1983–1984 *Diadema antillarum* mass mortality and severe overfishing of herbivorous fish [18,19], leading to a dramatic and persistent reduction in grazing pressure. In contrast, many Indo-Pacific reefs, especially those considered quasi-pristine, tend to maintain higher and more diverse populations of herbivorous fish, which are highly effective in controlling macroalgal growth [20,21]. Furthermore, varying intensities of other local anthropogenic stressors like eutrophication and coastal development have contributed to divergent trajectories of reef health and macroalgal abundance across these regions [22,23]. In light of these findings, Bruno et al. [24] argued that such observations, when considered alongside ecological theory and the impacts of over-harvesting in systems like the Caribbean, indicate that historically, macroalgal biomass may have been higher than previously assumed. This emerging paradigm challenges the traditional understanding, proposing that under natural conditions, macroalgae and corals can and do coexist in a competitive equilibrium. A growing body of research suggests that interactions between these two groups are not always strictly competitive but can be neutral or even beneficial in certain contexts, e.g., [25,26,27]. For example, studies in subtropical coral-dominated reefs have shown that macroalgae can thrive in close association with corals without causing visible stress or overgrowth, with fine-scale habitat features often dictating the nature of these relationships [28]. This dynamic balance, largely mediated by herbivory as previously documented, indicates that the relationship between macroalgae and corals extends beyond simple categorizations like competition [2].

Chemical ecological experiments, in particular, have started to shed light on the beneficial as well as detrimental effects of macroalgal chemicals on early coral life stages [29,30,31], as reviewed for the brown algal genus *Lobophora* in Vieira [27] and Vieira et al. [32]. Furthermore, macroalgae have been observed to harbor free-living *Symbiodinium* (or dinoflagellates) [33], potentially playing a role in maintaining symbiont diversity during ecological shifts [34]. Recognizing that ecological communities are shaped by long evolutionary histories, macroalgal–coral interactions inherently involve more complex dynamics than mere competition. To explore and decipher the nature of these interactions, proper baseline documentation of these natural interactions is imperative. However, the lack of baseline data on the qualitative and quantitative biological interactions between macroalgae and corals hampers our ability to unravel these intricate relationships. Despite the potential ecological importance of these interactions, few studies have systematically investigated Macroalgal–Coral Interaction (MCI) diversity, association typologies, and spatial distribution in healthy reefs, e.g., [16,17,28,35,36].

This study aims to address the existing knowledge gap by documenting natural MCI in healthy to mildly disturbed reefs within the second-largest reef system globally, situated in the southern Pacific Ocean, specifically in the lagoon of New Caledonia. The primary objectives are to (1) quantify and (2) qualitatively characterize the occurrences of MCI involving the most conspicuous macroalgae and corals. Additionally, the study seeks to (3) investigate the spatial distribution of MCI in coral-dominated habitats, thereby establishing a foundational baseline. These objectives are pivotal for future research endeavors seeking to unravel the intricate nature of these interactions and will provide essential baseline data necessary to comprehend the complexity of MCI, offering valuable insights for the ongoing monitoring and preservation of reef health.

## 2. Materials and Methods

### 2.1. Survey Area

**Figure 1:**
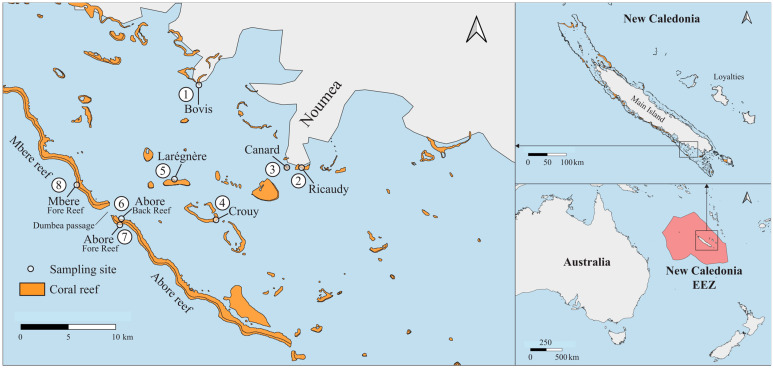
Map indicating survey sites in the South West lagoon of New Caledonia.

The present research was conducted in the South West Lagoon of New Caledonia (SWLNC) in April 2012 (Figure 1). The SWLNC is a semi-enclosed lagoon approximately 120 km long and 20 km wide with an average depth of 17.5 m (Figure 1). This region is characterized by a tropical climate that influences its oceanographic conditions. Seawater temperatures in the SWLNC typically range from approximately 22 °C in the austral winter (July–August) to 28–30 °C in the austral summer (January–February). Seasonal temperature variation in the lagoon is notably greater than its interannual variation, with temperatures in bays generally higher during summer and lower during winter compared to the open lagoon [37]. Salinity generally averages around 35.5 practical salinity units (PSU) [38] exhibiting minor seasonal fluctuations primarily influenced by rainfall and terrestrial runoff, especially during the wet season [37]. Both seasonal and interannual variations in salinity are amplified nearshore areas [37]. Long-term monitoring efforts in the region have indicated a slight warming trend of ~0.6 °C observed in the twentieth century coral Sr/Ca–SST record [39], although this is accompanied by notable decadal fluctuations [40].

**Figure 2:**
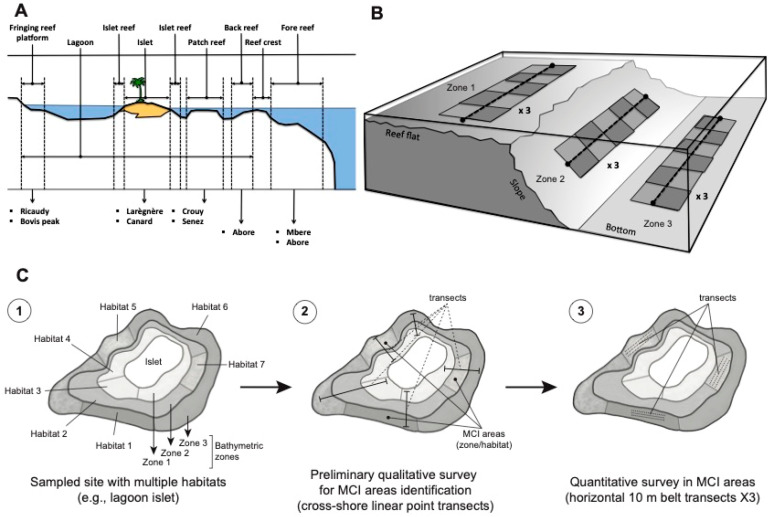
Schematic representation of the type and bathymetric zones investigated, and sites selection during this study along a shore to fore reef gradient (**A**,**B**). Illustration of the exploration of macroalgal–coral interaction (MCI) for example in a lagoon islet reef displaying three zones and varied habitats (**C**) (1), the MCI areas were identified through cross-shore linear point transects during the preliminary survey (2), then MCI were quantified in the selected MCI areas using triplicate belt transects in the selected habitats during the quantitative survey (3).

In this study, a macroalgal–coral interaction (MCI) was operationally defined as a direct and sustained contact between a coral colony and an adjacent macroalga. Direct contact encompassed any instance where the macroalgal thallus was physically touching or visibly growing over the living tissue or skeletal surface of the coral. ‘Sustained’ contact was specifically determined by the observation of stable physical entanglement or persistent pressure of the alga against the coral, indicating a non-incidental or ephemeral interaction that could potentially lead to competition for space or light. Fleeting or ephemeral contacts, where the alga was merely brushing against the coral due to water movement without firm entanglement or direct pressure, were not counted. The survey of MCI was conducted across eight reefs, spanning a gradient from the shoreline to the fore reef (Figure 1, Table 1). Various reef types, such as fringing reefs, islet reefs, patch reefs, back reefs, and fore reefs (illustrated in Figure 2), were included in the investigation. These reefs exhibited diverse anthropogenic influences, providing a diverse spectrum of environmental conditions for analysis (Table 1).

While MCI occur in various coral reef habitats, including macroalgal beds, seagrass beds, sandy bottoms, and coral fields, our focus in this study was on coral-dominated habitats. The classification of habitats was determined by considering both reef geomorphology and coral benthic cover, as outlined in Table 2. Reef geomorphology was deconstructed into two hierarchical levels, encompassing reef type and reef zonation (see Figure 2). This distinction was made because different reef types might exhibit analogous zonation patterns and benthic covers, emphasizing the need for a nuanced exploration of these factors.

A comprehensive selection comprising twenty-six distinct habitats was made based on considerations of benthic cover and coral structure, encompassing the majority of reef habitat diversity within the SWLNC (Table 3).

### 2.2. Data Collection

#### 2.2.1. Preliminary Qualitative Survey

A preliminary survey was undertaken to achieve two key objectives: (1) a qualitative evaluation MCI within the study area, and (2) identification of habitats showcasing the most prominent MCI for subsequent quantitative assessments. This identification was achieved through visual assessment and rapid qualitative surveys, involving extensive inspection across various reef zones to pinpoint areas characterized by a high apparent density of macroalgal–coral contacts, significant instances of macroalgal overgrowth on coral colonies, and/or notable physical entanglement between macroalgae and corals. These observations enabled the targeted selection of sites where MCI were visually dominant and ecologically significant for detailed quantitative surveys.

Survey sites were strategically chosen using raw satellite imagery from Google Earth version 7.1.2.2041, using Landsat satellite images (http://www.earth.google.com [accessed on 26 April 2012]), to target representative locations (Figure 2A). During this initial survey, Linear Point Intercept (LPI) transects, following the methodology outlined by English et al. [41], were implemented along a cross-shore section, extending from the sandy bottom to the reef (Figure 2A–C). MCI assessments were conducted at 50 cm intervals along the LPI transects, which could span up to 300 m. For islets and patch reefs, LPI transects were conducted in the four main cardinal directions. In the case of fringing, back, and fore reefs, four transects were conducted, considering diverse wind exposures (e.g., leeward and windward). A total of 36 LPI transects were conducted across the study area. Close-up photographs of each MCI were captured during this preliminary survey. Identifications of corals and macroalgae were carried out up to the genus level.

Quantification of MCI in selected areas using triplicate belt transects across diverse habitats during the quantitative survey.

#### 2.2.2. Quantitative Survey

Benthic cover and quantitative assessments of MCI were conducted using 10 m belt transects, following the methodology outlined by English, Wilkinson and Baker [41], in areas identified during the preliminary survey as exhibiting conspicuous MCI (Figure 2C). Transects were deployed in triplicate per area, aligned parallel to the isobaths (i.e., horizontal transects) and positioned 10 m apart. This resulted in a total of 78 transects across various habitats.

Within each transect, 50 × 50 cm quadrats were systematically positioned 20 times consecutively on both the left and right sides along a defined line. Photographs were captured directly above each quadrat using a Lumix Panasonic digital camera (12 megapixels), mounted on a photoquadrat framer (i.e., tetrapod). Additionally, close-up pictures of various MCI within the transect were documented. This comprehensive approach provided detailed data for both benthic cover and MCI across the study area.

### 2.3. Typology of Macroalgal–Coral Associations

Based on visual observations during both surveys, we categorized and defined types of interactions between macroalgae and corals, presented in the results section.

### 2.4. MCI Inventory and Abundance Assessment

The quantification of MCI and the determination of relative benthic cover, encompassing corals, macroalgae, and other macrobenthic organisms, were conducted utilizing a stratified random point count method CPCe [42] based on images captured during horizontal transects.

Individual photographs were subdivided into 16 equal squares, and each square was assigned a single random point within its borders. Employing a circle with crosshairs, featuring a diameter of 150 pixels as the data point object shape, the area beneath the crosshairs was systematically assessed. The specific feature under the crosshairs was recorded using the code identifiers available in Supplementary File S1. In instances where the crosshairs intersected with a coral or macroalga, the presence of macroalgae or corals in direct contact within the circle was documented. Subsequently, the abundance of specific interactions was calculated based on the recorded data. This approach allowed for a robust evaluation of MCI occurrences and facilitated the quantitative analysis of interaction patterns.

### 2.5. Interaction Index Calculation

To explore the relationship between algal abundance and their ecological role, we calculated an interaction index for 12 key macroalgal genera at each site. Raw quadrat data were first separated into two distinct datasets: one containing abundance counts for each genus (number of points per transect), and another containing interaction counts (number of observed inter-species contacts). We then aggregated these counts to the site level by summing the total abundance and total interactions for each genus at a given site. The interaction index was then calculated for each genus at each site using the formula: Interaction Index = Total Abundance/Total Interactions. This metric allowed us to evaluate the level of species-level interaction normalized by the genera’s overall abundance. The data processing and analysis were conducted in R, using the readr, dplyr, and stringr packages.

### 2.6. Estimation of MCI Richness

To gauge the richness of MCI in the SWLNC, we employed three sample-based richness estimators: the incidence-based coverage estimator ICE [43], Chao 2 richness estimators Chao 2 [44], and the Jackknife 1 first-order Jackknife richness estimator Jack 1 [45]. These species richness estimators are commonly utilized to infer species richness from a random sub-sample of individuals drawn from a larger sample.

ICE provides a distinction between frequently and infrequently occurring species during analysis. Jack 1 does not differentiate based on species frequency and relies on the count of MCI found only once. Chao 2, on the other hand, takes into account the number of unique units and duplicates. The MCI sample-based data served as the foundation for calculating these three richness estimators using EstimateS version 9.1.0 [46]. This approach facilitated a comprehensive assessment of the MCI richness within the study area.

To further assess the adequacy of our sampling effort, we complemented the MCI accumulation analysis with richness accumulation curves for the individual coral and macroalgal genera observed. This approach, using species-based data, provided an additional measure of sampling completeness. Richness accumulation curves were generated in R version 4.2.0 [47] using the iNEXT version 2.0.20 package [48] for both coral and macroalgal genera abundance data. The curves were used to visualize the relationship between sampling effort and the number of genera observed, confirming that our sampling adequately captured the diversity of these groups in the study area.

### 2.7. Spatial Patterns of MCI

To unveil the spatial patterns of MCI across diverse habitats in the SWLNC, we conducted a multiple correspondence analysis (MCA) [49]. MCA serves as an extension of correspondence analyses, tailored for scenarios involving multiple variables in categorical data. It offers a robust method for scrutinizing the relationships among various categorical dependent variables.

In this analysis, we focused on two biological variables: the presence of macroalgae and corals, and three environmental variables: reef type, reef zonation, and benthic cover. For the MCA, we selectively considered the six most prevalent macroalgae and coral species. This selection was made to focus on the most ecologically dominant interactions and ensure robust statistical inference, as less abundant species had a low frequency of occurrence that would preclude meaningful statistical comparisons of their interaction patterns. While this approach allows for a detailed examination of the most prevalent interactions shaping reef communities, it is important to acknowledge that it may not fully capture rare or cryptic interactions involving less abundant species, which could nonetheless play specialized ecological roles. The MCA was executed using FactoMineR [50] in the R statistical environment [47]. This comprehensive approach enabled us to decipher the intricate spatial patterns of MCI across the studied habitats.

## 3. Results

### 3.1. Typology of Macroalgal–Coral Associations

**Figure 3:**
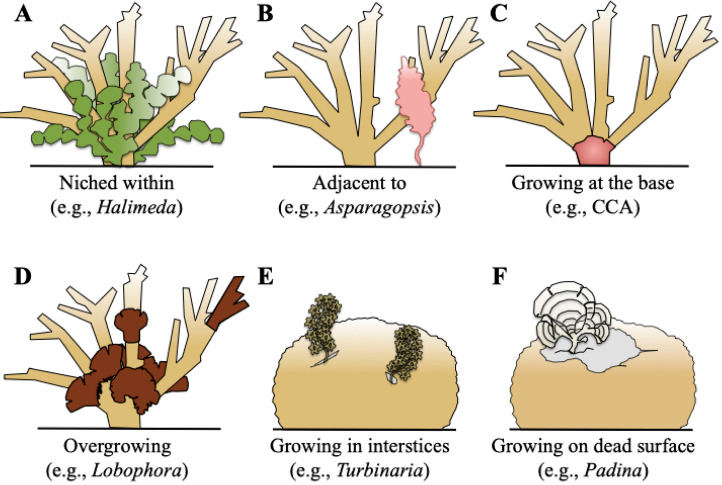
A schematic representation of the six types of macroalgal–coral associations found in coral-dominated habitats within the South West Lagoon of New Caledonia. The classifications are: (**A**) “Niched within”, where macroalgae are nestled within coral branches. (**B**) “Adjacent to”, with macroalgae growing in close proximity but not in direct contact with live coral. (**C**) “Growing at the base”, where macroalgae are attached to the substrate directly at the base of a coral colony. (**D**) “Overgrowing live coral tissue”, illustrating a physical spread of macroalgae onto the coral. (**E**) “Growing in dead interstices”, showing macroalgae occupying crevices within dead coral structures. (**F**) “Growing on dead surfaces”, where macroalgae are attached to the outer surfaces of dead coral. CCA: Crustose Coralline Algae.

**Figure 4:**
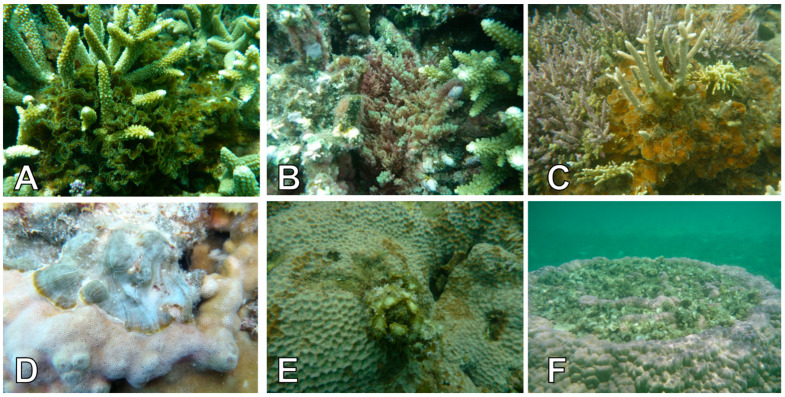
Photographic illustrations of the six types of macroalgal–coral associations observed in the coral-dominated habitats of the South West Lagoon of New Caledonia. (**A**) *Lobophora rosacea* niched within the branches of *Acropora* sp. (**B**) *Asparagopsis taxiformis* adjacent to a live *Acropora* sp. colony. (**C**) *Lobophora monticola* growing at the base of *Acropora muricata*. (**D**) *Lobophora obscura* overgrowing *Montipora* sp. (**E**) *Turbinaria ornata* growing within the interstices of *Porites* sp. (**F**) *Padina* sp. growing on dead surface of *Porites* sp. coral head.

Our observations revealed six distinct types of macroalgal–coral associations. Each type provides insights into the spatial configurations and apparent relationships between these organisms at the time of survey: (1) Niched among (Figure 3A and Figure 4A): Exemplified by *Lobophora*, *Halimeda*, *Dictyota*, and *Hypnea*, macroalgae were observed nestled within the complex branching structures of coral colonies. This configuration suggests a close spatial proximity where macroalgae utilize the coral habitat for support or refuge; (2) Adjacent to (Figure 3B and Figure 4B): Illustrated by *Asparagopsis*, macroalgae were recorded growing in direct proximity to live coral colonies, with their thalli extending towards or alongside the coral, but without clear, direct contact or apparent tissue interaction at the observed interface. This indicates a close spatial relationship that maintains a boundary between the two organisms; (3) Growing at the base (Figure 3C and Figure 4C): Instances involving *Lobophora* and crustose coralline algae (CCA) showcased macroalgae attached and growing from the hard substrate directly at the base of live coral colonies. This configuration suggests a supportive and stable foundation provided by the underlying reef structure, immediately adjacent to the coral; (4) Overgrowing live coral tissue (Figure 3D and Figure 4D): Illustrated by *Lobophora*, this typology describes instances where macroalgal thalli were observed physically spreading over and in direct contact with apparent live coral tissue. While this morphology can indicate a competitive interaction, in the absence of temporal data, it represents the observed state where macroalgae appeared to extend onto live coral. It is important to note that this observation alone does not definitively confirm active tissue invasion or mortality caused by the macroalgae, and could, in some cases, reflect colonization onto areas of prior, unobserved tissue mortality or temporary contact. Further temporal studies would be required to ascertain the dynamic nature and precise outcome of such interactions; (5) Growing in (dead) interstices (Figure 3E and Figure 4E): Exemplified by *Turbinaria*, this association revealed macroalgae growing within the intricate spaces and crevices of dead coral structures. This highlights their opportunistic use of existing dead reef structures, providing insights into ecological niches for macroalgal growth in areas not occupied by live coral tissue; (6) On Dead Surfaces (Figure 3F and Figure 4F): Illustrated by *Padina*, macroalgae were observed growing on dead coral surfaces, showcasing a commensal interaction where the coral provides substratum and protection for the macroalga.

This typology classification enhances our understanding of the multifaceted relationships between macroalgae and corals, providing a nuanced perspective on the ecological dynamics within coral-dominated habitats.

### 3.2. MCI Diversity

In the preliminary survey, we visually documented 43 MCI involving 10 coral genera (*Acropora*, *Galaxea*, *Montipora*, *Pavona*, *Pocillopora*, *Porites*, *Seriatopora*, *Stylophora*, *Turbinaria*) and 16 macroalgal genera (*Asparagopsis*, *Amphiroa*, *Caulerpa*, *Ceratodictyon*, *Chaetomorpha*, *Chlorodesmis*, *Colpomenia*, *Dictyota*, *Galaxaura*, *Halimeda*, *Hydroclathrus*, *Hypnea*, *Liagora*, *Lobophora*, *Padina*, *Sargassum*), along with crustose coralline algae (CCA) and turf algae (Table S1). However, several of these interactions were infrequently observed during the prospection period (Figure S1), and macroalgae growing underneath branching corals may have been overlooked despite our efforts.

**Figure 5:**
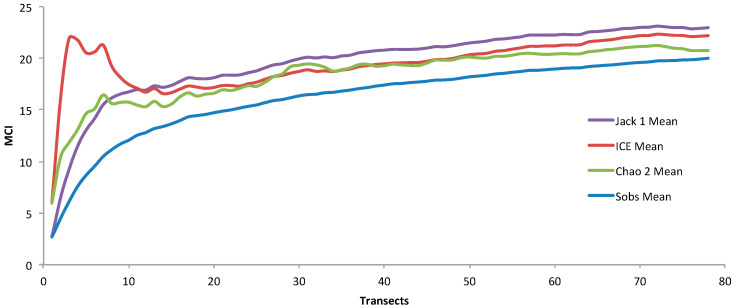
Observed and estimated richness accumulation curves for macroalgal–coral interactions (MCI) in the South West Lagoon of New Caledonia. The solid blue line shows the observed number of distinct MCI (Sobs). The other colored lines represent non-parametric sample-based richness estimators: the Incidence-based Coverage Estimator (ICE, red), Chao 2 (green), and first-order Jackknife (Jack 1, purple). Richness is shown as a function of the number of transects.

To estimate the potential diversity of MCI in the SWLNC, we applied three species richness estimators: incidence-based coverage estimator (ICE), Jackknife 1 (Jack 1), and Chao 2. These estimators converged on similar values (Figure 5), suggesting an expected richness between 21 ± 1.4 (Chao 2; mean ± standard deviation) and 23 ± 1.7 (Jack 1; mean ± standard deviation) interactions, slightly higher than the observed diversity of 20 interactions (Sobs) based on quantitative survey data. This indicates that our sampling effort adequately captured a substantial portion of the MCI diversity in the studied area.

To address concerns regarding sampling effort, we generated richness accumulation curves for the coral and macroalgal genera separately (Figures S2 and S3). Both curves approach an asymptote, indicating that our sampling adequately captured the majority of the genera present at the study sites. This finding, combined with the convergence of the MCI richness estimators (Figure 5), confirms the robustness of our sampling methodology for assessing both interaction richness and the underlying taxonomic diversity.

### 3.3. MCI Abundance and Patterns

Utilizing data from the preliminary survey, we estimated the prevalence of MCI at a reef scale, constituting an average of 16.4% of the benthic cover in coral-dominated habitats. In habitats where MCI were particularly conspicuous, the interaction frequency between benthic reef macroalgae and scleractinian corals reached up to 70%, averaging 30% within surveyed belt transects. The relative abundance of these interactions across different reef types, zonations, and benthic cover categories is detailed in Figure S4. It is crucial to note that these percentages are specific to sites intentionally chosen for their high MCI abundance and cannot be extrapolated to estimate macroalgal presence across all coral-dominated habitats within New Caledonian reefs.

**Figure 6:**
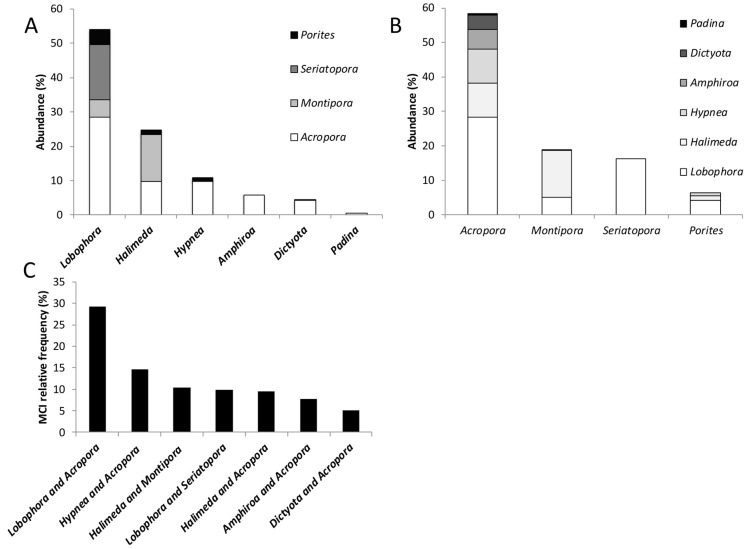
Macroalgal–coral interactions in the South West lagoon of New Caledonia. (**A**) Relative abundance (in percentage) of dominant coral taxa (*Lobophora*, *Halimeda*, *Hypnea*, *Amphiroa*, *Dictyota*, *Padina*) showing the proportional contribution of various coral genera (*Porites*, *Seriatopora*, *Montipora*, *Acropora*) to the overall abundance within each dominant coral group. (**B**) Abundance (in percentage) of specific coral genera (*Acropora*, *Montipora*, *Seriatopora*, *Porites*) showing the proportional contribution of different macroalgae (*Padina*, *Dictyota*, *Amphiroa*, *Hypnea*, *Halimeda*, *Lobophora*) interacting with them. (**C**) Relative frequency of different macroalgal–coral interaction types (e.g., *Lobophora* and *Acropora*, *Hypnea* and *Acropora*, *Halimeda* and *Seriatopora*, etc.) based on the Macroalgal–Coral Interaction (MCI) index.

*Lobophora* emerged as the most prevalent macroalgal representative, found in 47% of all MCI, followed by *Halimeda* (20%) and *Hypnea* (9%) (Figure 6A). Among scleractinian corals, *Acropora* dominated in direct contact with macroalgae (Figure 6B), contributing to 61% of all MCI, followed by *Montipora* (19%), *Seriatopora* (13%), and *Porites* (5%). While macroalgae displayed a preference for branching, columnar, and digitate corals, some genera such as *Lobophora* and crustose coralline algae were also found at the basal part or on dead surfaces (e.g., *Padina*, *Chlorodesmis*) of large, massive, and encrusting corals (e.g., *Porites*, *Montipora*). These findings unveil specific patterns in the distribution and interaction of macroalgae and corals, providing valuable insights into the dynamics of these interactions within coral-dominated habitats.

Macroalgal–coral interactions, constituting more than 5% of all recorded interactions across transects, are visually depicted in Figure 6C. Notably, the *Lobophora–Acropora* interaction emerged as the most prevalent and conspicuous MCI, encompassing 29% of the total interactions. Following closely, *Hypnea–Acropora* accounted for 15%, while *Halimeda–Montipora*, *Lobophora–Seriatopora*, and *Halimeda–Acropora* each contributed approximately 10% to the observed interactions. It is noteworthy that *Lobophora–Seriatopora*, despite representing a significant proportion of all MCI, was exclusively observed in the barrier reef, where *Seriatopora* tends to flourish. These findings shed light on the specific MCI that play a substantial role in the coral-dominated habitats of the SWLNC.

### 3.4. Spatial Patterns of MCI

**Figure 7:**
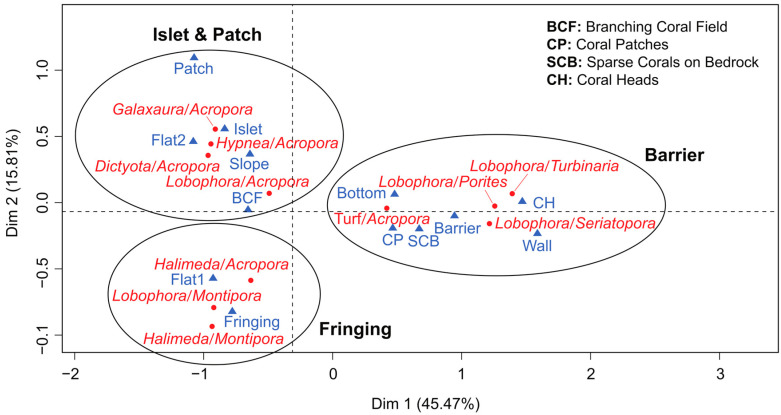
Multiple correspondence analysis map of macroalgal–coral interactions (MCI) and habitat variables (reef type, reef zonation, and benthic cover). BCF: Branching Coral Field. CP: Coral Patch. SCB: Scarce Coral on Bedrock. CH: Coral Head.

To unveil the intricate relationships between MCI and habitat variables such as reef type, reef zonation, and benthic cover, a comprehensive multiple correspondence analysis (MCA) was conducted. The MCA results elucidated the close associations between specific MCI and distinct habitats. The first dimension of the MCA, accounting for 45% of the variability, notably segregates the barrier reef from other reef types (Figure 7). Furthermore, the second dimension, contributing to 16% of the variability, predominantly distinguishes the fringing reefs from the islet reefs (Figure 7).

Specific MCI, including *Lobophora–Seriatopora*, *Lobophora–Turbinaria*, *Lobophora–Porites*, and turf–*Acropora*, were predominantly observed in the inner barrier, characterized by sparse coral in bedrock and on walls. On the other hand, *Halimeda–Acropora*, *Halimeda–Montipora*, and *Lobophora–Montipora* exhibited a higher prevalence in flat fringing reefs. Remarkably, the occurrence of MCI appeared independent of anthropogenic disturbance, emphasizing the robust nature of these interactions across diverse reef habitats.

### 3.5. Algal Interaction Index vs. Abundance

The supplementary analysis revealed no clear linear relationship between a genus total abundance and its interaction index across the study sites (Figure S1). A high abundance did not consistently correspond to a high interaction index. Notably, certain genera with relatively low abundance demonstrated a disproportionately high interaction index at specific sites.

Among the specific genera analyzed, *Lobophora* had a moderate interaction index despite being the most prevalent and abundant macroalga. In contrast, *Halimeda* and *Hypnea* displayed high interaction indices relative to their respective abundances. *Amphiroa* and *Dictyota* exhibited the lowest interaction indices, consistent with their low overall abundances in the study area. This varied relationship between abundance and interaction index among genera reinforces that there is no consistent linear relationship between the two metrics across all sites.

## 4. Discussion

By extensively documenting Macroalgal–Coral Interactions (MCI) within coral-dominated habitats spanning various reefs, ranging from fringing to fore reefs, across the South West lagoon of New Caledonia (SWLNC), our study provides crucial insights of these biological interactions in relatively undisturbed reef systems. Our primary objectives were to quantify and characterize MCI, investigate their spatial distribution, and establish a baseline for future research.

### 4.1. Diversity, Richness, and Abundance of Macroalgal–Coral Interactions

This study employed a two-phased approach to capture both the diversity and abundance of MCI. The preliminary qualitative survey identified a high diversity of 43 unique interactions, including rare or cryptic interactions. The second phase, employing systematic belt transects and photoquadrats, documented 20 interactions, reflecting a focus on statistically robust, common occurrences rather than total potential diversity. Species richness estimators (Chao 2, Jackknife 1, ICE) converged on an expected diversity of 21–23 interactions, confirming that the quantitative survey captured the majority of dominant interactions.

Overall, MCI constituted a relatively small proportion of benthic cover in coral-dominated habitats (16.4% on average) consistent with observations from other healthy reefs where herbivory and other ecological controls maintain a competitive equilibrium between corals and macroalgae [2,21,51]. These findings highlight that while MCI are present in New Caledonian reefs, their abundance remains moderate, challenging the assumption that macroalgal interactions are rare in undisturbed coral reef systems.

### 4.2. Specificity and Spatial Patterns of Interactions

MCI occurrences were not random but reflected strong affinities between particular macroalgal and coral genera. *Lobophora* was the most frequent macroalgal interactant, occurring in 47% of all MCI, followed by *Halimeda* (20%) and *Hypnea* (9%). Among corals, *Acropora* dominated (61%), followed by *Montipora* (19%) and *Seriatopora* (13%). This disproportionate involvement of certain genera reflects both the susceptibility of some coral species to close algal associations and the preferential settlement of particular macroalgae on certain coral microhabitats.

The prevalence of an interaction was not directly proportional to the abundance of the interacting taxon. While *Lobophora* was abundant, it displayed only a moderate Interaction Index, indicating that its frequent encounters with corals primarily reflect ubiquity rather than specific association. Conversely, *Halimeda* and *Hypnea* exhibited high Interaction Indices relative to their abundance, suggesting specialized life history traits favoring coral-associated habitats. Rare genera such as *Amphiroa* and *Dictyota* showed low Interaction Indices, reinforcing that abundance alone does not predict interaction frequency.

Habitat type strongly structured these associations. Multiple correspondence analysis (MCA) revealed reef type as the primary factor influencing MCI distribution. For instance, the *Lobophora hederacea–Seriatopora caliendrum*/*S. hystrix* association was restricted to barrier reefs, aligning with the ecological preferences of both interactants [52,53,54]. In contrast, *Halimeda–Acropora* associations were most frequent in fringing reefs, highlighting that the spatial distribution of macroalgae, in addition to coral presence, determines MCI patterns.

### 4.3. Typologies of Associations and Coralgal Biotic Interaction Compass (CBIC)

**Figure 8:**
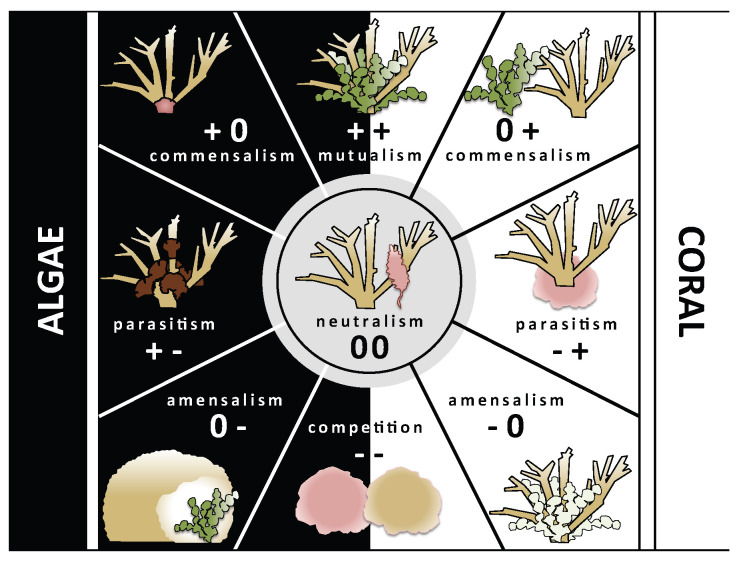
Coralgal Biotic Interaction Compass. Interactions with beneficial or neutral effects for the algae on the left side (black background), and for the coral on the right side (white background). +: positive effect, 0: neutral effect, -: negative effect.

We identified six typologies of macroalgal–coral associations in the southwest lagoon of New Caledonia, from algae “niched within” coral branches to those “growing on dead surfaces” (Figure 3 and Figure 4). These typologies describe the modes of physical association between corals and macroalgae, i.e., how the two organisms are spatially configured. However, typologies alone do not determine the ecological consequences of the association. To capture the nature of interactions—that is, what each partner gains or loses—we introduce the Coralgal Biotic Interaction Compass (CBIC; Figure 8). The CBIC classifies associations according to their net effect on each interactant, ranging from mutualism (negative for both) to competition (negative for both), with intermediate categories including commensalism, amensalism, neutralism (no effect on either partner), and parasitism.

The ways in which macroalgae and corals can benefit from these associations are diverse. Macroalgae mainly gain benefits from corals in the form of substrata, reduced hydrodynamic stress, and structural refuges, enabling persistence where grazing pressure might otherwise exclude them [28,55]. Corals may benefit indirectly from macroalgae, for example, via harboring free-living *Symbiodinium* that may be available to the coral [33,56], or by reduced predation on juvenile corals by acting as deterrents or “sacrificial buffers” against corallivorous fishes (i.e., algal cover can draw fish grazing pressure away from juvenile corals, indirectly shielding them, though this protection may be offset by competitive disadvantages) [57,58]. Conversely, negative outcomes may occur when associations impair growth, reproduction, or survival, either through shading, abrasion, pathogen transfer, or allelopathic effects [13].

The same typology can correspond to different CBIC categories depending on the balance of benefits and costs. For example, *Lobophora* “niched within” *Acropora* branches may represent commensalism (alga gains refuge, coral unaffected), amensalism (alga benefits, stressed), or parasitism (alga exploits coral tissue, e.g., *Lobophora hederacea* with *Seriatopora caliendrum* [54]). Conversely, corals themselves can act as parasites, such as coral larvae settling on crustose coralline algae [59]. *Padina* “growing on dead surfaces” of massive *Porites* would typically be commensal, with the alga benefiting from substratum while the coral is unaffected. Only in a few cases, such as macroalgae directly “overgrowing” live coral tissue, does the typology strongly imply a negative outcome for the coral, which may take the form of parasitism (alga exploiting live coral tissue as a net benefit) or competition (both coral and alga losing fitness through direct resource conflict). These examples emphasize that the presence of macroalgae in close proximity does not necessarily equate to coral stress or decline.

It is important to note that our dataset represents snapshot observations. While typologies can be robustly identified from a single survey, assigning interactions to CBIC categories requires temporal monitoring or experimental testing to determine whether coral and macroalgal fitness is affected over time. Moreover, the same typology may shift across categories depending on environmental conditions. In healthy, high-herbivory reefs, macroalgae associated with corals often act as neutral or commensal partners, while under stress (bleaching, disease, reduced herbivory), the same associations may transition toward parasitism or competition.

The combined use of typologies and the CBIC therefore provides a conceptual framework for disentangling the complexity of MCI. Typologies describe the form of association, while the CBIC categorizes the function. Together, they underscore that macroalgal-coral associations are not inherently antagonistic but can shift across a continuum of outcomes shaped by ecological context and disturbance history.

### 4.4. The Role of Coral Architecture and Genus-Specific Traits

Spatial patterns of MCI are strongly influenced by coral architecture. Branching and columnar corals provide microhabitats for algal settlement, refuge from herbivory, and reduced dislodgement due to lower water flow [25,60,61]. De Carvalho and Villaca [28] showed that microhabitat features, rather than species identity alone, govern these interactions, with structural complexity facilitating algal settlement without competitive displacement.

Macroalgae such as *Lobophora*, *Halimeda*, and *Hypnea* preferentially associate with structurally complex corals in the SWLNC, suggesting that these interactions are often commensal or protective mutualisms, rather than inherently harmful. Even when *Lobophora* overgrows *Seriatopora* colonies, this may reflect opportunistic response following prior disturbances such as localized bleaching [52,53,54,55]. Herbivory experiments indicate that physical refuge within coral structures may be a more important defense for algae than chemical deterrents [62]. This highlights the dual role of coral architecture in both facilitating algal recruitment and protecting established algae from grazing pressure.

**Figure 9:**
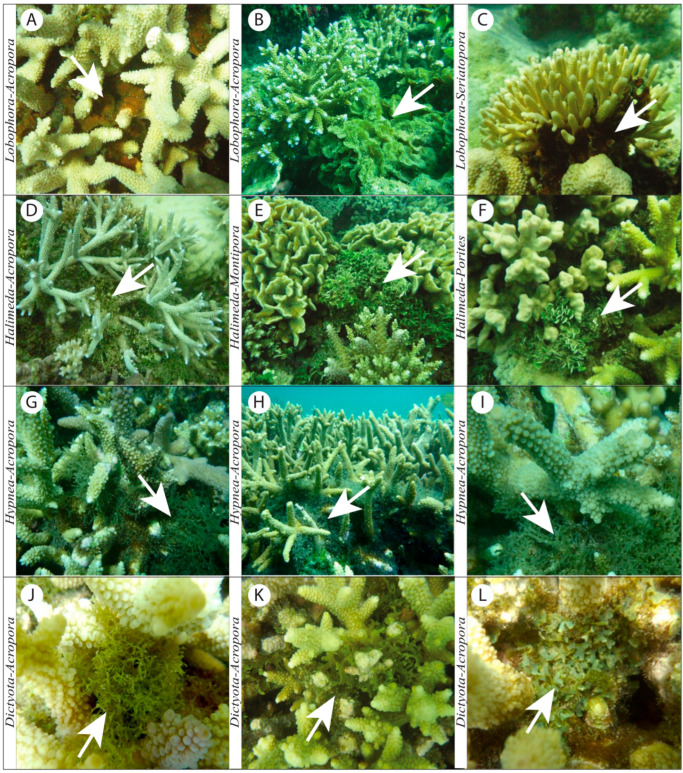
Photographic illustrations of dominant macroalgal–coral associations in the coral-dominated habitats of the South West Lagoon of New Caledonia. (**A**,**B**). *Lobophora–Acropora* association, often found on dead basal parts of branching colonies. (**C**) *Lobophora–Seriatopora*, an association noted on the barrier reef during this study, where *Lobophora hederacea* can overgrow colonies. (**D**) *Halimeda–Acropora*, an articulated calcareous green alga often nestled within the intricate branches of coral colonies. (**E**) *Halimeda–Montipora* association. (**F**) *Halimeda–Porites* association, where the alga is typically found at the base of the colony. (**G**–**I**) *Hypnea–Acropora* association, where the alga forms low-lying clumps nestled beneath the coral canopy with little negative impact. (**J**–**L**) *Dictyota–Acropora*, with the alga restricted to the bases of colonies, appearing as prostrate or low-erect thalli.

In New Caledonia, several *Lobophora* species commonly grow in close association with coral colonies, typically at the base or creeping onto skeletons [52,53,54,63] (Figure 9A–C). These species rarely cause extensive bleaching in the SWLNC reefs and are predominantly associated with dead basal parts of branching colonies [63]. The relationship spans a continuum from seemingly neutral or commensal associations to aggressive overgrowth, as observed for *L. hederacea* on *Seriatopora caliendrum* and *S. hystrix*. However, our observations provide only a temporal snapshot, limiting firm conclusions about the exact nature of these interactions.

Extensive research has examined the role of *Lobophora* in reef regime shifts, which are dramatic transitions from coral- to algal-dominated states. Vieira [27] synthesized these studies, highlighting that while *Lobophora* is widespread, only a few species actively compete with or negatively affect corals. Most negative interactions, including large-scale blooms, occur in disturbed reef systems and are often symptomatic of underlying stressors, such as bleaching, reduced herbivory, or nutrient enrichment, rather than characteristic of healthy ecosystems.

Taxonomic studies have revealed remarkable *Lobophora* diversity in New Caledonia, exceeding 30 species, each with distinct ecological affinities [53]. Despite this diversity, only a subset (~<10%) actively associates with corals, including *L. dimorpha*, *L. hederacea*, *L. monticola*, *L. rosacea*, and *L. undulata*. Most species inhabit dead basal coral skeletons and rarely induce bleaching, suggesting primarily neutral, commensal, or potentially mutualistic associations.

Evidence for species-specific associations between *Lobophora* and corals is increasingly documented beyond New Caledonia. In Thailand, certain local *Lobophora* species exhibit preferential interactions with specific coral hosts [64], and in the Caribbean, similar patterns have been observed [65]. Complementing these findings, Puk et al. [66] investigated the cryptic diversity of *Lobophora* in Palau and found that specific species assemblages were strongly structured by environmental drivers such as wave exposure, depth, and herbivore biomass. Although their study did not focus on direct MCI, it introduces the important concept of ecological specialists and generalists within the genus. Taken together, these studies suggest that the species-specific interactions observed in New Caledonia may reflect a combination of ecological specialization: some *Lobophora* species are specialists on particular coral hosts, while others may be specialists on specific environmental conditions rather than particular biotic partners.

A particularly notable—and apparently antagonistic—interaction was observed on the barrier reef (Abore back reef), where *Lobophora hederacea* exhibited a strong associational preference for branching *Seriatopora* colonies (*S. caliendrum* and *S. hystrix*) [54] (Figure 9C). This ranged from basal colonization to substantial overgrowth of the colony. While temporal monitoring is lacking, both in our study and in Vieira, Payri and De Clerck [54], additional evidence suggests that this pattern may reflect a response to environmental stress rather than inherent competitive dominance. Experimental work indicates that *S. caliendrum* and *S. hystrix* are particularly sensitive to thermal stress, with even moderate anomalies [67,68,69] capable of weakening coral defenses and facilitating algal settlement. Notably, during 2010–2011, New Caledonia experienced above-average sea surface temperatures associated with a cold La Niña event, potentially leading to localized bleaching of *Seriatopora* colonies [70]. Comparable observations from the Keppel Islands (Great Barrier Reef, Australia) following the 2006 bleaching events further support the view that algal overgrowth often represents post-disturbance opportunism rather than primary competitive exclusion [71].

Herbivory experiments suggest limited chemical defense among New Caledonian *Lobophora*, highlighting the importance of ecological and growth traits in providing refuge [58]. Close associations with corals may thus function as a form of protective mutualism, even when competitive interactions exist.

Regional context strongly mediates *Lobophora*’s ecological role. In the Indo-Pacific, *Lobophora* remains part of the background benthos, exerting localized and reversible effects on corals [64,65,66]. By contrast, in the Caribbean, *L. declerckii* (previously referred to as *L. variegata*) proliferates on degraded reefs, forming persistent mats that suppress coral recovery [67,68]. These contrasts underscore the importance of reef health and disturbance history in determining whether *Lobophora* acts as a benign associate or dominant competitor.

Further insights from herbivory experiments [62] suggest that chemical defenses among New Caledonian *Lobophora* species are limited, as there was little interspecific variation in susceptibility to grazing. This finding leads to the hypothesis that the species’ ecological or growth habit may serve as a more effective defense against herbivory. In this context, the close association with coral colonies may provide a physical refuge for the alga, protecting it from grazing pressure. This interpretation presents a nuanced perspective on the relationship, suggesting that while the association can be competitive, it may also be a form of protective mutualism.

The regional context of *Lobophora* is critical for interpreting its ecological role. In the Indo-Pacific, *Lobophora* is widespread on coral-dominated reefs but typically remains part of the background benthos, exerting only localized and reversible effects on corals [63,72,73]. By contrast, in the Caribbean, the taxon long referred to as “*L. variegata*” (but more accurately corresponding to *L. declerckii*; [see 66] has proliferated on degraded reefs, where it contributes to persistent coral–algal phase shifts [74,75]. The ecological success and broad distribution of *L. declerckii* in the Greater Caribbean appear linked to its capacity to form extensive, persistent mats that suppress coral recovery. This sharp contrast between ocean basins underscores how regional context—particularly reef health and disturbance history—mediates whether *Lobophora* functions as a background associate or a dominant competitor.

In the South West Lagoon of New Caledonia, coral-dominated reefs, *Halimeda* was frequently observed at the base of colonies or nestled within the intricate branches of *Montipora* and *Acropora* (Figure 9D–F). This articulated, calcareous green alga is among the dominant calcareous macroalgae on tropical reefs and plays a dual ecological role: contributing substantially to reef carbonate production and providing structural habitat [76,77]. Its high Interaction Index relative to abundance indicates that its ecological influence is disproportionate to its cover, reflecting a specialized role in close macroalgal-coral associations. From the alga’s perspective, branching corals offer a refuge from herbivory, with the structural complexity of *Acropora* particularly effective at sheltering *Halimeda*, resulting in carbonate production nearly three times higher inside coral branches than in exposed habitats [61]. These coral microhabitats may also provide indirect benefits to corals: *Halimeda* aggregations have been shown to harbor free-living *Symbiodinium* spp., potentially acting as a source of symbionts for aposymbiotic coral recruits [33,78]. However, *Halimeda* can also act as a competitor under disturbed conditions. In Palk Bay (India), a reef system stressed by disease, sponge overgrowth, overfishing, and tidal exposure, *Halimeda* was observed contacting over a third of surveyed coral colonies, predominantly *Porites* (57%), followed by *Favites* (28%) and *Acropora* (26%) [79]. Dense clusters can abrade coral tissues, release allelopathic compounds that impair coral recovery, and serve as reservoirs for pathogenic bacteria [80,81,82]. Combined with high growth rates and strong chemical and structural defenses against herbivores, these traits make *Halimeda* a formidable post-disturbance competitor [83]. The extent of its impact varies with coral morphology and local herbivore communities [84,85]. Thus, *Halimeda* exemplifies the context-dependent nature of MCI: when sheltered within branching corals, it benefits from reduced grazing and may indirectly support coral recruits, but under disturbed or degraded conditions, it can shift to become a strong competitor, potentially affecting coral fitness and recovery.

In the South West Lagoon of New Caledonia, *Hypnea* was most often observed as low-lying clumps nestled among *Acropora* branches, generally beneath the coral canopy (Figure 9G–I). These corticated red algae appeared to exert little negative impact on their coral hosts. Our field observations are consistent with experimental work on the Great Barrier Reef, where mats of *Hypnea pannosa* in contact with *Porites cylindrica* caused minimal tissue damage or growth inhibition [81,86]. The alga’s delicate, filamentous morphology likely limits shading, abrasion, and smothering, allowing it to persist in close association with corals. Nonetheless, more recent work suggests interactions may not always be neutral: in a controlled experiment, direct contact between *H. pannosa* and *P. cylindrica* induced measurable oxidative stress in coral tissues [87]. Overall, while *Hypnea* is a common element of Indo-Pacific reef assemblages, studies explicitly examining its ecological relationships with corals remain scarce compared to the more intensively studied *Lobophora* and *Halimeda*.

*Dictyota* species were generally restricted to the bases of *Acropora* colonies, in the SWLNC, forming prostrate or low-erect thalli beneath the branch canopy (Figure 9J–L). Their occurrence was discrete, without obvious signs of coral tissue damage or overgrowth. Globally, however, *Dictyota*–coral interactions reveal sharp regional contrasts. In the Caribbean, multiple *Dictyota* species act as potent competitors, suppressing coral recruitment and survival via physical overgrowth and allelopathic metabolites [88,89], with numerous experimental studies documenting their capacity to inhibit coral resilience on already degraded reefs. In contrast, in the Indo-Pacific, *Dictyota* is abundant but typically forms part of the background algal community, rarely dominating benthic cover or directly harming corals [90]. Our observations from New Caledonia fit this latter pattern, emphasizing that the ecological role of *Dictyota* is highly context-dependent: competitive and often detrimental in the Caribbean’s long-disturbed reef systems, e.g., [91,92,93], but generally benign in the Indo-Pacific, where reefs remain structurally more intact and herbivory pressure higher.

### 4.5. Human Influence and MCI

Our study did not specifically investigate the effects of human influence on MCI, but we did document these interactions across a range of sites with varying levels of protection. As shown in Table 1, MCI were recorded in locations designated as Natural or Integral Marine Reserves (e.g., Bovis, Canard, Abore, Laregnere) as well as in areas with no formal restrictions (e.g., Ricaudy, Crouy, Mbere). This observation suggests that these interactions are not confined to reefs heavily impacted by human activity; instead, they appear to be an intrinsic and natural part of the dynamics of New Caledonia’s coral reef systems. While previous studies have shown that high nutrient levels and reduced herbivory can increase macroalgal abundance in human-impacted areas [1,11], our data indicate that the occurrence of MCI itself is not exclusively a symptom of anthropogenic degradation. A more comprehensive understanding of the relationship between human impact and MCI would require further analyses with more detailed data on specific human influence metrics for each site.

### 4.6. MCI Spatial Patterns: Signals of Long-Term Coevolution in Coral–Macroalgal–Herbivore Assemblages

Our SWLNC dataset shows that MCI are widespread but patterned, with repeatable pairings such as *Lobophora–Seriatopora* on barrier reefs and *Halimeda–Acropora* on fringing reefs, and with non-random habitat affinities. These observations reinforce a growing body of work indicating that close macroalgal–coral associations are normal features of intact reefscapes—not merely symptoms of degradation—and that their ecological outcomes depend on context, partners, and history rather than on competition alone. Early syntheses challenged the “seaweeds = degradation” paradigm by documenting substantial macroalgal presence on reefs considered healthy and lightly impacted, such as the Line Islands, the Northwestern Hawaiian Islands, and Howland and Baker Islands, and by urging a reassessment of baseline conditions for “how much macroalgae is natural” [16,17,24]. In parallel, broader conceptual work emphasized that macroalgal–coral encounters span a continuum—from commensal or protective associations to outright competition—mediated by herbivory, hydrodynamics, and microhabitat structure rather than by dyadic antagonism alone [2,13,25,35].

Several lines of evidence from our study align with this evolutionarily informed perspective. First, the architecture of coral colonies appears to channel these associations. Branching and columnar morphologies, particularly *Acropora*, *Montipora* and *Seriatopora*, hosted disproportionately high numbers of macroalgal contacts. This is consistent with the idea that complex colonies generate microhabitats that reduce flow and dislodgement, provide refuges from herbivory, and offer shaded surfaces within the canopy for algal settlement [25,28,60]. Such architecture-driven mechanisms help explain why some macroalgae exhibit high Interaction Indices despite modest cover, and why the same algal species may appear benign when nestled within coral branches yet act as competitors when expanding opportunistically after disturbance [61,79].

Second, our results highlight the importance of host and habitat filtering rather than ubiquity in shaping these interactions. The restriction of *Lobophora hederacea–Seriatopora* interactions to barrier reefs mirrors patterns documented elsewhere: *Lobophora* shows strong species-level ecological partitioning in New Caledonia, with more than 30 species but only a minority consistently engaging corals [53,54,62,63,65,66]. Similarly, our finding that *Halimeda–Acropora* interactions were concentrated in fringing reefs agrees with studies showing that structurally complex corals can both shelter and modulate *Halimeda* dynamics [61]. Moreover, *Halimeda* can oscillate between neutrality or even positive roles, such as providing habitat for free-living *Symbiodinium* that may subsidize coral recruits, and more antagonistic roles under conditions of stress or reduced grazing [33,78]. These cases illustrate that both algal and coral identity, combined with local habitat features, act as dual filters that determine the frequency and outcome of their interactions.

A third dimension is the triadic role of herbivory. Decades of experiments and syntheses have shown that grazing pressure, exerted by browsing and scraping guilds, largely determines whether macroalgae persist as background associates or become competitive dominants [12,19,21,84,85]. Our observation that canopy-forming corals sheltered macroalgae otherwise vulnerable to herbivory is consistent with the hypothesis that seaweeds exploit coral architecture as associational refuges, while corals may derive incidental benefits from the presence of macroalgae, including reduced parrotfish damage to juvenile corals or “sacrificial buffer” effects [57,58]. The prevalence of neutral or commensal outcomes in our coral-dominated SWLNC sites therefore reflects a herbivore-mediated equilibrium, which can be shifted toward antagonism by thermal anomalies, nutrient enrichment, or herbivore depletion [2,14,35].

Finally, these associations must be understood within their deep evolutionary context. Corals have coexisted with both calcareous and fleshy algae for millions of years, and groups such as *Halimeda* and coralline algae have been integral not only to modern reef sediment production and carbonate budgets [76,77] but also to reef framework construction in geological time [94] and contemporary contributions to reef building that can rival corals themselves [95]. Selection over geological timescales has thus shaped macroalgal traits that exploit coral architecture and hydrodynamics, coral traits that mitigate the costs of incidental neighbors, and herbivore behaviors that maintain coexistence. Our detection of repeatable MCI in relatively undisturbed systems echoes this long history and emphasizes that such interactions are intrinsic to reef functioning.

Together, these observations position the spatial structuring of MCI as more than a descriptive pattern: it provides a framework for mechanistic research on the benefits and costs of coral–macroalgal associations. By coupling system-level observations, as presented here, with experimental tests of reciprocal benefits in canopy microhabitats, species-resolved studies of *Lobophora* ecology, and cross-habitat comparisons, future work can refine our understanding of when and how these interactions shift along the continuum from neutral or beneficial to competitive. Our SWLNC results therefore do not overturn earlier interpretations but rather add support—especially from coral-dominated habitats—to the view that MCI are intrinsic components of healthy reef systems [2,24]. The same coral–algal pairs can manifest as neutral, commensal, or antagonistic depending on partner identity, habitat, and disturbance history, which is precisely why spatial patterns of interaction, interpreted through the lenses of coral architecture and herbivore dynamics, offer a valuable window into the evolutionary assembly and present-day functioning of reef communities.

## 5. Conclusions

This study represents the first comprehensive assessment of macroalgal–coral interactions (MCI) in the South West Lagoon of New Caledonia, part of the world’s largest lagoon and second-largest barrier reef complex. By documenting the diversity, abundance, and spatial distribution of MCI across multiple reef types and habitats, it provides a critical ecological baseline for one of the most extensive reef systems in the Indo-Pacific. The interactions recorded here—moderate in abundance and largely non-aggressive in nature—reinforce observations from other Indo-Pacific reefs that such associations are a normal feature of undisturbed coral-dominated ecosystems. Beyond cataloguing these interactions, this study introduces a dual framework combining interaction typologies and the Coralgal Biotic Interaction Compass (CBIC), offering a standardized approach for describing both the structure and potential ecological roles of coral–algal relationships. Future research should extend these observations through temporal monitoring and finer taxonomic resolution, as species-specific interactions (e.g., *Lobophora hederacea–Seriatopora caliendrum*/*hystrix*) may underlie patterns masked at the genus level. Expanding similar surveys across Indo-Pacific and Atlantic reefs will be essential for understanding how local ecological filters and environmental change shape the diversity and dynamics of macroalgal–coral interactions.

## Figures and Tables

**Table 1:** Survey sites in the South West lagoon of New Caledonia.

Site #	Site Name	Reef Type	Distance to Shore (km)	Human Influence
1	Bovis	Fringing reef	0.1	Natural reserve
2	Ricaudy	Fringing reef	0.1	No restrictions
3	Canard	Islet reef	1	Natural reserve
4	Crouy	Patch reef	10	No restrictions
5	Laregnere	Islet reef	12	Natural reserve
6	Abore	Back reef	18	Integral marine reserve
7	Abore	Fore reef	19	Integral marine reserve
8	Mbere	Fore reef	21	No restrictions

**Table 2:** Hierarchical classification of coral-dominated habitats based on reef type, reef zonation, and benthic cover.

Reef Type	Reef Zonation	Benthic Cover
Fringing	Flat	Branching coral field
Islet	Slope	Sparse corals and bedrock
Patch	Wall	Coral heads
Back reef	Bottom	Coral patches
Fore reef	Slope	Coral patches

**Table 3:** Habitats selected to quantify MCI in the southwest lagoon of New Caledonia.

Habitat #	Habitat Code	Location	Reef Type	Zonation	Benthos	Depth (m)	Additional Note
1	ABO_0001	Abore	Patch reef	Slope	BCF	2–5	Patch reef
2	ABO_0002	Abore	Patch reef	Flat	SCBR	0–2	Patch reef
3	ABO_0003	Abore	Back reef	Wall	SCBR	2–5	Spur and groove
4	ABO_0004	Abore	Back reef	Flat	SCBR	0–2	Spur
5	ABO_0005	Abore	Back reef	Bottom	BCF	5–10	Groove
6	ABO_0006	Abore	Back reef	Bottom	CH	5–10	Groove
7	ABOOUT	Abore	Fore reef	Slope	SCBR	10–15	-
8	BOV_0001	Bovis	Fringing reef	Bottom	CH	5–10	-
9	BOV_0002	Bovis	Fringing reef	Slope	BCF	2–5	-
10	BOV_0003	Bovis	Fringing reef	Flat	BCF	0–2	-
11	BOV_0004	Bovis	Fringing reef	Flat	SBC	0–2	-
12	CAN_0001	Canard	Islet reef	Bottom	BCF	5–10	Windward
13	CAN_0003	Canard	Islet reef	Slope	BCF	2–5	Windward
14	CAN_0004	Canard	Islet reef	Flat	SCBR	0–2	Windward
15	CAN_0009	Canard	Islet reef	Slope	BCF	2–5	Leeward
16	CAN_0014	Canard	Islet reef	Flat	SPCB	0–2	Leeward
17	CRO_0003	Crouy	Patch reef	Flat	BCF	0–2	Leeward
18	CRO_0004	Crouy	Patch reef	Flat	BCF	0–2	Leeward
19	CRO_0010	Crouy	Patch reef	Flat	BCF	0–2	Windward
20	RIC_0002	Ricaudy	Fringing reef	Flat/slope	BCF	0–2	Windward
21	LAR_0001	Laregnere	Islet reef	Bottom	CH	5–10	Leeward
22	LAR_0002	Laregnere	Islet reef	Slope	BCF	2–5	Leeward
23	LAR_0003	Laregnere	Islet reef	Flat	BCF	0–2	Outside islet lagoon
24	LAR_0007	Laregnere	Islet reef	Flat	BCF	0–2	Inside islet lagoon
25	LAR_0010	Laregnere	Islet reef	Flat	SCBR	0–2	-
26	MBEOUT	Mbere	Fore reef	Slope	SCBR	10–15	-

BCF: Branching Coral Field; SCBR: Sparse Corals and Bedrock; CH: Coral Heads.

## Data Availability

Data are contained within the article.

## Supplementary Materials

The following supporting information can be downloaded at: https://www.mdpi.com/article/10.3390/biology14101419/s1, Supplementary File S1: Code identifiers used for benthic substrate classification and analysis in CPCe; Table S1: Macroalgal-coral interaction (MCI) observed during the preliminary qualitative survey; Figure S1: Relative abundance of macroalgal-coral interaction (MCI), in percentage, in the studied area in the southwest lagoon of New Caledonia; Figure S2: Richness accumulation curves for macroalgal genera recorded in the South West Lagoon of New Caledonia. Curves show observed richness (Sobs, black) and non-parametric estimators Bootstrap (red), Chao 2 (blue), first-order Jackknife (Jack 1, green), and second-order Jackknife (Jack 2, purple), plotted as a function of the number of transects (sampling units). All curves tend toward an asymptote, confirming that sampling effort was sufficient to capture the majority of macroalgal generic diversity; Figure S3: Richness accumulation curves for coral genera recorded in the South West Lagoon of New Caledonia. Curves show observed richness (Sobs, black) and non-parametric estimators Bootstrap (red), Chao 2 (blue), first-order Jackknife (Jack 1, green), and second-order Jackknife (Jack 2, purple), plotted as a function of the number of transects (sampling units). The curves approach a plateau, indicating that sampling effort adequately represented coral generic diversity across sampled habitats; Figure S4. Macroalgal–coral interactions relative abundance, in percentage, in the South West lagoon of New Caledonia, per habitat levels (A) Reef type, (B) Reef zonation, (C) Benthic cover.

## Abbreviations

The following abbreviations are used in this manuscript:

MCI	Macroalgal–coral interaction
SWLNC	Southwest lagoon of New Caledonia

## References

[1] Done T.T., Ogden J.J., Wiebe W., Rosen B., Mooney H.A., Cushman J.H., Medina E., Sala O.E., Schulze E.D. (1996). Biodiversity and ecosystem function of coral reefs. Functional Roles of Biodiversity: A Global Perspective.

[2] Vieira C., Payri C., De Clerck O. (2016). A fresh look at macroalgal-coral interactions: Are macroalgae a threat to corals?. Perspect. Phycol..

[3] Fong P., Paul V.J., Dubinsky Z., Stambler N. (2011). Coral reef algae. Coral Reefs: An Ecosystem in Transition.

[4] Morrissey J. Primary productivity of coral reef bsenthic macroalgae. Proceedings of the 5th International Coral Reef Congress.

[5] Tano S.A., Eggertsen M., Wikström S.A., Berkström C., Buriyo A., Halling C. (2017). Tropical seaweed beds as important habitats for juvenile fish. Mar. Freshw. Res..

[6] Stamski R.E., Field M.E. (2006). Characterization of sediment trapped by macroalgae on a Hawaiian reef flat. Estuar. Coast. Shelf Sci..

[7] Evans R., Wilson S., Field S., Moore J. (2014). Importance of macroalgal fields as coral reef fish nursery habitat in north-west Australia. Mar. Biol..

[8] Puk L.D., Ferse S.C., Wild C. (2016). Patterns and trends in coral reef macroalgae browsing: A review of browsing herbivorous fishes of the Indo-Pacific. Rev. Fish Biol. Fish..

[9] Verges A., Bennett S., Bellwood D.R. (2012). Diversity among macroalgae-consuming fishes on coral reefs: A transcontinental comparison. PLoS ONE.

[10] Côté I., Gill J., Gardner T., Watkinson A. (2005). Measuring coral reef decline through meta-analyses. Philos. Trans. R. Soc. B Biol. Sci..

[11] Schutte V.G., Selig E.R., Bruno J.F. (2010). Regional spatio-temporal trends in Caribbean coral reef benthic communities. Mar. Ecol. Prog. Ser..

[12] Hay M.E. (1981). Herbivory, algal distribution, and the maintenance of between-habitat diversity on a tropical fringing reef. Am. Nat..

[13] McCook L., Jompa J., Diaz-Pulido G. (2001). Competition between corals and algae on coral reefs: A review of evidence and mechanisms. Coral Reefs.

[14] Folke C., Carpenter S., Walker B., Scheffer M., Elmqvist T., Gunderson L., Holling C. (2004). Regime shifts, resilience, and biodiversity in ecosystem management. Annu. Rev. Ecol. Evol. Syst..

[15] Rasher D.B., Hay M.E. (2010). Seaweed allelopathy degrades the resilience and function of coral reefs. Commun. Integr. Biol..

[16] Vroom P.S., Braun C.L. (2010). Benthic composition of a healthy subtropical reef: Baseline species-level cover, with an emphasis on algae, in the Northwestern Hawaiian Islands. PLoS ONE.

[17] Vroom P.S., Musburger C.A., Cooper S.W., Maragos J.E., Page-Albins K.N., Timmers M.A. (2010). Marine biological community baselines in unimpacted tropical ecosystems: Spatial and temporal analysis of reefs at Howland and Baker Islands. Biodivers. Conserv..

[18] Jackson J., Donovan M., Cramer K., Lam V. (2014). Status and Trends of Caribbean Coral Reefs: 1970–2012.

[19] Edwards C.B., Friedlander A.M., Green A., Hardt M., Sala E., Sweatman H., Williams I.D., Zgliczynski B., Sandin S.A., Smith J.E. (2014). Global assessment of the status of coral reef herbivorous fishes: Evidence for fishing effects. Proc. R. Soc. B Biol. Sci..

[20] Wu P., Wang T., Liu Y., Li C., Xiao Y., Xu S., Han T., Lin L., Quan Q. (2022). Differences of macroalgal consumption by eight herbivorous coral reef fishes from the Xisha Islands, China. Front. Mar. Sci..

[21] Fabricius K.E., Crossman K., Jonker M., Mongin M., Thompson A. (2023). Macroalgal cover on coral reefs: Spatial and environmental predictors, and decadal trends in the Great Barrier Reef. PLoS ONE.

[22] Morrow K.M., Ritson-Williams R., Ross C., Liles M.R., Paul V.J. (2012). Macroalgal extracts induce bacterial assemblage shifts and sublethal tissue stress in Caribbean corals. PLoS ONE.

[23] United States Environmental Protection Agency (EPA) U.S. Coral Reefs. https://www.epa.gov/coral-reefs/us-coral-reefs.

[24] Bruno J.F., Precht W.F., Vroom P.S., Aronson R.B. (2014). Coral reef baselines: How much macroalgae is natural?. Mar. Pollut. Bull..

[25] Bennett S., Vergés A., Bellwood D. (2010). Branching coral as a macroalgal refuge in a marginal coral reef system. Coral Reefs.

[26] Steinberg P.D., De Nys R. (2002). Chemical mediation of colonization of seaweed surfaces. J. Phycol..

[27] Vieira C. (2020). *Lobophora*–coral interactions and phase shifts: Summary of current knowledge and future directions. Aquat. Ecol..

[28] De Carvalho L.L., Villaca R.C. (2021). Effect of fine-scale habitat differences on algal colonisation in a coral-dominated subtropical reef. An. Acad. Bras. Ciências.

[29] Birrell C.L., McCook L.J., Willis B.L., Harrington L. (2008). Chemical effects of macroalgae on larval settlement of the broadcast spawning coral *Acropora millepora*. Mar. Ecol. Prog. Ser..

[30] Paul V.J., Kuffner I.B., Walters L.J., Ritson-Williams R., Beach K.S., Becerro M.A. (2011). Chemically mediated interactions between macroalgae *Dictyota* spp. and multiple life-history stages of the coral *Porites astreoides*. Mar. Ecol. Prog. Ser..

[31] Fong J., Lim Z.W., Bauman A.G., Valiyaveettil S., Liao L.M., Yip Z.T., Todd P.A. (2019). Allelopathic effects of macroalgae on *Pocillopora acuta* coral larvae. Mar. Environ. Res..

[32] Vieira C., Gaubert J., De Clerck O., Payri C., Culioli G., Thomas O.P. (2015). Biological activities associated to the chemodiversity of brown algae belonging to the genus *Lobophora* (Dictyotales, Phaeophyceae). Phytochem. Rev..

[33] Porto I., Granados C., Restrepo J.C., Sanchez J.A. (2008). Macroalgal-associated dinoflagellates belonging to the genus *Symbiodinium* in Caribbean reefs. PLoS ONE.

[34] LaJeunesse T. (2002). Diversity and community structure of symbiotic dinoflagellates from Caribbean coral reefs. Mar. Biol..

[35] Barott K.L., Williams G.J., Vermeij M.J., Harris J., Smith J.E., Rohwer F.L., Sandin S.A. (2012). Natural history of coral-algae competition across a gradient of human activity in the Line Islands. Mar. Ecol. Prog. Ser..

[36] Haas A., El-Zibdah M., Wild C. (2010). Seasonal monitoring of coral–algae interactions in fringing reefs of the Gulf of Aqaba, Northern Red Sea. Coral Reefs.

[37] Ouillon S., Douillet P., Fichez R., Panché J.-Y. (2005). Enhancement of regional variations in salinity and temperature in a coral reef lagoon, New Caledonia. Comptes Rendus Geosci..

[38] Pacific S.W. (1997). ENSO signals in the vicinity of New Caledonia. Oceanol. Acta.

[39] DeLong K.L., Quinn T.M., Taylor F.W. (2007). Reconstructing twentieth-century sea surface temperature variability in the southwest Pacific: A replication study using multiple coral Sr/Ca records from New Caledonia. Paleoceanography.

[40] Quinn T.M., Crowley T.J., Taylor F.W., Henin C., Joannot P., Join Y. (1998). A multicentury stable isotope record from a New Caledonia coral: Interannual and decadal sea surface temperature variability in the southwest Pacific since 1657 AD. Paleoceanography.

[41] English S.S., Wilkinson C.C., Baker V.V. (1994). Survey Manual for Tropical Marine Resources.

[42] Kohler K.E., Gill S.M. (2006). Coral Point Count with Excel extensions (CPCe): A Visual Basic program for the determination of coral and substrate coverage using random point count methodology. Comput. Geosci..

[43] Chao A., Lee S.-M. (1992). Estimating the number of classes via sample coverage. J. Am. Stat. Assoc..

[44] Chao A. (1987). Estimating the population size for capture-recapture data with unequal catchability. Biometrics.

[45] Burnham K.P., Overton W.S. (1979). Robust estimation of population size when capture probabilities vary among animals. Ecology.

[46] Colwell R.K. (2013). EstimateS: Statistical Estimation of Species Richness and Shared Species from Samples. http://purl.oclc.org/estimates.

[47] R Core Team (2023). R: A Language and Environment for Statistical Computing.

[48] Hsieh T., Ma K., Chao A. (2020). iNEXT: Interpolation and extrapolation for species diversity (2.0.20). Comprehensive R Archive Network (CRAN).

[49] Greenacre M., Blasius J. (2006). Multiple Correspondence Analysis and Related Methods.

[50] Lê S., Josse J., Husson F. (2008). FactoMineR: An R package for multivariate analysis. J. Stat. Softw..

[51] Kopp D., Bouchon-Navaro Y., Louis M., Mouillot D., Bouchon C. (2010). Herbivorous fishes and the potential of Caribbean marine reserves to preserve coral reef ecosystems. Aquat. Conserv.-Mar. Freshw. Ecosyst..

[52] Vieira C. (2015). *Lobophora* Biotic Interactions and Diversification. Ph.D. Thesis.

[53] Vieira C., D’hondt S., De Clerck O., Payri C.E. (2014). Toward an inordinate fondness for stars, beetles and *Lobophora*? Species diversity of the genus *Lobophora* (Dictyotales, Phaeophyceae) in New Caledonia. J. Phycol..

[54] Vieira C., Payri C., De Clerck O. (2015). Overgrowth and killing of corals by the brown alga *Lobophora hederacea* (Dictyotales, Phaeophyceae) on healthy reefs in New Caledonia: A new case of the epizoism syndrome. Phycol. Res..

[55] Monismith S.G. (2007). Hydrodynamics of coral reefs. Annu. Rev. Fluid Mech..

[56] Venera-Ponton D.E., Diaz-Pulido G., Rodriguez-Lanetty M., Hoegh-Guldberg O. (2010). Presence of *Symbiodinium* spp. in macroalgal microhabitats from the southern Great Barrier Reef. Coral Reefs.

[57] Venera-Ponton D.E., Diaz-Pulido G., McCook L.J., Rangel-Campo A. (2011). Macroalgae reduce growth of juvenile corals but protect them from parrotfish damage. Mar. Ecol. Prog. Ser..

[58] Brooker R.M., Sih T.L., Dixson D.L. (2017). Contact with seaweed alters prey selectivity in a coral-feeding reef fish. Mar. Ecol. Prog. Ser..

[59] Tebben J., Motti C., Siboni N., Tapiolas D., Negri A., Schupp P., Kitamura M., Hatta M., Steinberg P.D., Harder T. (2015). Chemical mediation of coral larval settlement by crustose coralline algae. Sci. Rep..

[60] Brown K.T., Bender-Champ D., Kubicek A., van der Zande R., Achlatis M., Hoegh-Guldberg O., Dove S.G. (2018). The dynamics of coral-algal interactions in space and time on the southern Great Barrier Reef. Front. Mar. Sci..

[61] Castro-Sanguino C., Lovelock C., Mumby P.J. (2016). The effect of structurally complex corals and herbivory on the dynamics of *Halimeda*. Coral Reefs.

[62] Vieira C., Stenger P.-L., Moleana T., De Clerck O., Payri C. (2019). Limited interspecific variation in grazing susceptibility of the brown alga *Lobophora* to herbivory. J. Exp. Mar. Biol. Ecol..

[63] Vieira C., Thomas O.P., Culioli G., Genta-Jouve G., Houlbreque F., Gaubert J., De Clerck O., Payri C.E. (2016). Allelopathic interactions between the brown algal genus *Lobophora* (Dictyotales, Phaeophyceae) and scleractinian corals. Sci. Rep..

[64] Klomjit A., Vieira C., Mattos F.M., Sutthacheep M., Sutti S., Kim M.-S., Yeemin T. (2022). Diversity and ecology of *Lobophora* species associated with coral reef systems in the western Gulf of Thailand, including the description of two new species. Plants.

[65] Vieira C., Morrow K.M., D’Hondt S., Camacho O., Engelen A.H., Payri C., De Clerck O. (2020). Diversity, ecology, biogeography and evolution of the prevalent brown algal genus *Lobophora* in the Greater Caribbean sea, including the description of five new species. J. Phycol..

[66] Puk L.D., Vieira C., Roff G., De Clerck O., Mumby P.J. (2020). Cryptic diversity in the macroalgal genus *Lobophora* (Dictyotales) reveals environmental drivers of algal assemblages. Mar. Biol..

[67] Seveso D., Montano S., Strona G., Orlandi I., Galli P., Vai M. (2016). Hsp60 expression profiles in the reef-building coral *Seriatopora caliendrum* subjected to heat and cold shock regimes. Mar. Environ. Res..

[68] Seveso D., Montano S., Strona G., Orlandi I., Galli P., Vai M. (2014). The susceptibility of corals to thermal stress by analyzing Hsp60 expression. Mar. Environ. Res..

[69] Moriarty T., Leggat W., Heron S.F., Steinberg R., Ainsworth T.D. (2023). Bleaching, mortality and lengthy recovery on the coral reefs of Lord Howe Island. The 2019 marine heatwave suggests an uncertain future for high-latitude ecosystems. PLoS Clim..

[70] Hasson A., Delcroix T., Boutin J., Dussin R., Ballabrera-Poy J. (2014). Analyzing the 2010–2011 La Niña signature in the tropical Pacific sea surface salinity using in situ data, SMOS observations, and a numerical simulation. J. Geophys. Res. Ocean..

[71] Diaz-Pulido G., McCook L.J., Dove S., Berkelmans R., Roff G., Kline D.I., Weeks S., Evans R.D., Williamson D.H., Hoegh-Guldberg O. (2009). Doom and boom on a resilient reef: Climate change, algal overgrowth and coral recovery. PLoS ONE.

[72] Diaz-Pulido G., McCook L.J. (2004). Effects of live coral, epilithic algal communities and substrate type on algal recruitment. Coral Reefs.

[73] Jompa J., McCook L.J. (2002). The effects of nutrients and herbivory on competition between a hard coral (*Porites cylindrica*) and a brown alga (*Lobophora variegata*). Limnol. Oceanogr..

[74] Hughes T.P. (1994). Catastrophes, phase shifts, and large-scale degradation of a Caribbean coral reef. Science.

[75] Mumby P.J. (2009). Phase shifts and the stability of macroalgal communities on Caribbean coral reefs. Coral Reefs.

[76] Granier B. (2012). The contribution of calcareous green algae to the production of limestones: A review. Geodiversitas.

[77] Multer H.G. (1988). Growth rate, ultrastructure and sediment contribution of *Halimeda incrassata* and *Halimeda monile*, Nonsuch and Falmouth Bays, Antigua, WI. Coral Reefs.

[78] Granados-Cifuentes C., Neigel J., Leberg P., Rodriguez-Lanetty M. (2015). Genetic diversity of free-living *Symbiodinium* in the Caribbean: The importance of habitats and seasons. Coral Reefs.

[79] Thinesh T., Jose P.A., Ramasamy P., Meenatchi R., Selvan K.M., Selvin J. (2019). Differential coral response to algae contact: *Porites* tissue loss, praise for *Halimeda* interaction at southeast coast of India. Environ. Sci. Pollut. Res..

[80] Nugues M.M., Smith G.W., Hooidonk R.J., Seabra M.I., Bak R.P. (2004). Algal contact as a trigger for coral disease. Ecol. Lett..

[81] Jompa J., McCook L.J. (2003). Coral-algal competition: Macroalgae with different properties have different effects on corals. Mar. Ecol. Prog. Ser..

[82] Hay M.E. (1997). The ecology and evolution of seaweed-herbivore interactions on coral reefs. Coral Reefs.

[83] Birrell C.L., McCook L.J., Willis B.L., Diaz-Pulido G.A. (2008). Effects of benthic algae on the replenishment of corals and the implications for the resilience of coral reefs. Oceanogr. Mar. Biol. Annu. Rev..

[84] Mantyka C.S., Bellwood D.R. (2007). Direct evaluation of macroalgal removal by herbivorous coral reef fishes. Coral Reefs.

[85] Mantyka C.S., Bellwood D.R. (2007). Macroalgal grazing selectivity among herbivorous coral reef fishes. Mar. Ecol. Prog. Ser..

[86] Jompa J., McCook L.J. (2003). Contrasting effects of turf algae on corals: Massive *Porites* spp. are unaffected by mixed-species turfs, but killed by the red alga *Anotrichium tenue*. Mar. Ecol. Prog. Ser..

[87] Yang H., Liu J., Huangfu M., Zhao H., Li Y., Zhou Y., Wang A., Chen R.-W., Li X. (2025). Physiological impairment and metabolic perturbations in *Porites cylindrica* induced by *Hypnea pannosa* contact. Mar. Environ. Res..

[88] Rasher D.B., Hay M.E. (2014). Competition induces allelopathy but suppresses growth and anti-herbivore defence in a chemically rich seaweed. Proc. R. Soc. B Biol. Sci..

[89] Bonaldo R.M., Hay M.E. (2014). Seaweed-coral Interactions: Variance in seaweed allelopathy, coral susceptibility, and potential effects on coral resilience. PLoS ONE.

[90] Birrell C.L. (2003). Influences of Benthic Algae on Coral Settlement and Post-Settlement Survival: Implications for the Recovery of Disturbed and Degraded Reefs. Ph.D. Thesis.

[91] Lirman D. (2001). Competition between macroalgae and corals: Effects of herbivore exclusion and increased algal biomass on coral survivorship and growth. Coral Reefs.

[92] Box S.J., Mumby P.J. (2007). Effect of macroalgal competition on growth and survival of juvenile Caribbean corals. Mar. Ecol. Prog. Ser..

[93] Titlyanov E., Yakovleva I., Titlyanova T. (2007). Interaction between benthic algae (*Lyngbya bouillonii*, *Dictyota dichotoma*) and scleractinian coral *Porites lutea* in direct contact. J. Exp. Mar. Biol. Ecol..

[94] Bosence D. (1983). Coralline algal reef frameworks. J. Geol. Soc..

[95] Cornwall C.E., Carlot J., Branson O., Courtney T.A., Harvey B.P., Perry C.T., Andersson A.J., Diaz-Pulido G., Johnson M.D., Kennedy E. (2023). Crustose coralline algae can contribute more than corals to coral reef carbonate production. Commun. Earth Environ..

