# Supplementary material for: Macroalgal–Coral Interactions in New Caledonia South West Lagoon: Diversity, Abundance, and Spatial Patterns
Source: Biology (Basel). 2025 Oct 15;14(10):1419. doi: 10.3390/biology14101419 (PMC12561486; doi:10.3390/biology14101419)
Supplement: Supplementary file 1 [file biology-14-01419-s001.zip › Supplementary_File S1.pdf]

biology

MDPI

Supplementary File S1. Code identifiers used for benthic substrate classification and analysis in CPCe.

DFDFDF

AFAFAF

8

"C", "Live coral",FF8000

"MA", "Macroalgae",00FF00

"MCI", "Macroalgae-coral interactions",FF80FF

"DC", "Dead coral",AFAFAF

"SE", "Sediment",FFFF80

"FA", "Fauna",FF0000

"ANG", "Angiosperm",008000

"TWS", "Tape, Wand, Shadow",808000

"Asp", "Asparagopsis", "MA"

"Cau", "Caulerpa", "MA"

"Cer", "Ceratodyction", "MA"

"Chl", "Chlorodesmis", "MA"

"Col", "Colpomenia", "MA"

"Dic", "Dictyota", "MA"

"Hal", "Halimeda", "MA"

"Hyd", "Hydroclathrus clathratus", "MA"

"Hyp", "Hypnea", "MA"

"Lob", "Lobophora", "MA"

"Pad", "Padina", "MA"

"RCM", "Red calcareous macroalgae", "MA"

"Sar", "Sargassum", "MA"

"Turb", "Turbinaria", "MA"

"Turf", "Turf", "MA"

"CA", "Caulerpa and acropora", "MCI"

"DA", "Dictyota and Acropora", "MCI"

"DMi", "Dictyota and Millepora", "MCI"

"DMo", "Dictyota and Montipora", "MCI"

"GA", "Galaxaura and Acropora", "MCI"

"DPc", "Dictyota and Pocillopora", "MCI"

"HA", "Halimeda and Acropora", "MCI"

"HMi", "Halimeda and Millepora", "MCI"

"HMo", "Halimeda and Montipora", "MCI"

"HPr", "Halimeda and Porites", "MCI"

"HyA", "Hypnea and Acropora", "MCI"

"HyPo", "Hypnea and Pocillopora", "MCI"

"HyPr", "Hypnea and Porites", "MCI"

"LA", "Lobophora and Acropora", "MCI"
"LMi", "Lobophora and Millepora", "MCI"
"LMo", "Lobophora and Montipora", "MCI"
"LPc", "Lobophora and Pocillopora", "MCI"
"LPr", "Lobophora and Porites", "MCI"
"LSc", "Lobophora and Seriatopora caliendrum", "MCI"
"LSh", "Lobophora and Seriatopora hystrix", "MCI"
"LT", "Lobophora and Turbinaria", "MCI"
"MAC", "Macralgae and Coral", "MCI"
"PA", "Padina and Acropora", "MCI"
"RA", "Red algae and acropora", "MCI"
"TA", "Turf and Acropora", "MCI"
"CeM", "Ceratodyction and Montipora", "MCI"
"HyM", "Hypnea and Acropora", "MCI"
"CeA", "Ceratodyction and Acropora", "MCI"
"SP", "Sponge", "FA"
"SC", "Soft coral", "FA"
"BC", "Branching Coral", "C"
"DCo", "Digitate coral", "C"
"EC", "Encrusting coral", "C"
"FC", "Foliose coral", "C"
"MC", "Massive coral", "C"
"Mil", "Millepora", "C"
"SMC", "Sub-massive coral", "C"
"TC", "Tabular coral", "C"
"CR", "Coral rubbles", "DC"
"DC", "Dead coral, Bleached coral", "DC"
"BR", "Bedrock", "SE"
"SD", "Sand", "SE"
"SHA", "Shadow", "TWS"
"SEA", "Seagrass", "ANG"
