# Supplementary material for: Macroalgal–Coral Interactions in New Caledonia South West Lagoon: Diversity, Abundance, and Spatial Patterns
Source: Biology (Basel). 2025 Oct 15;14(10):1419. doi: 10.3390/biology14101419 (PMC12561486; doi:10.3390/biology14101419)
Supplement: Supplementary file 1 [file biology-14-01419-s001.zip › Supplementary Figures and Table.pdf]

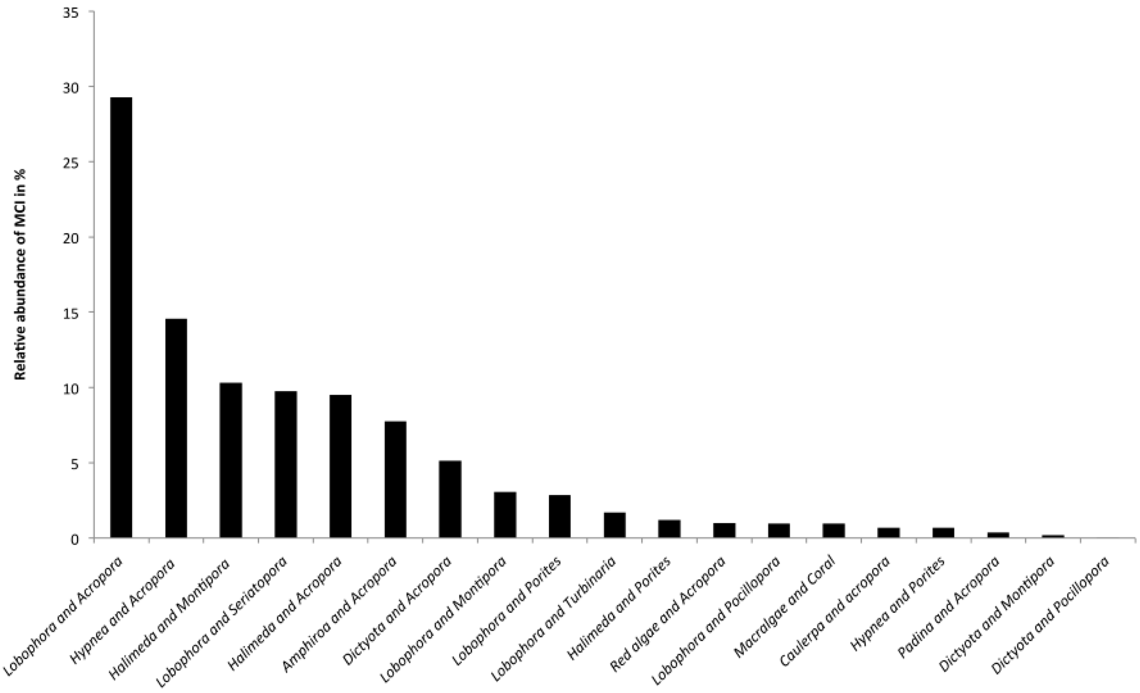


**Figure S1.** Relative abundance of macroalgal–coral interaction (MCI), in percentage, in the studied area in the South West Lagoon of New Caledonia.

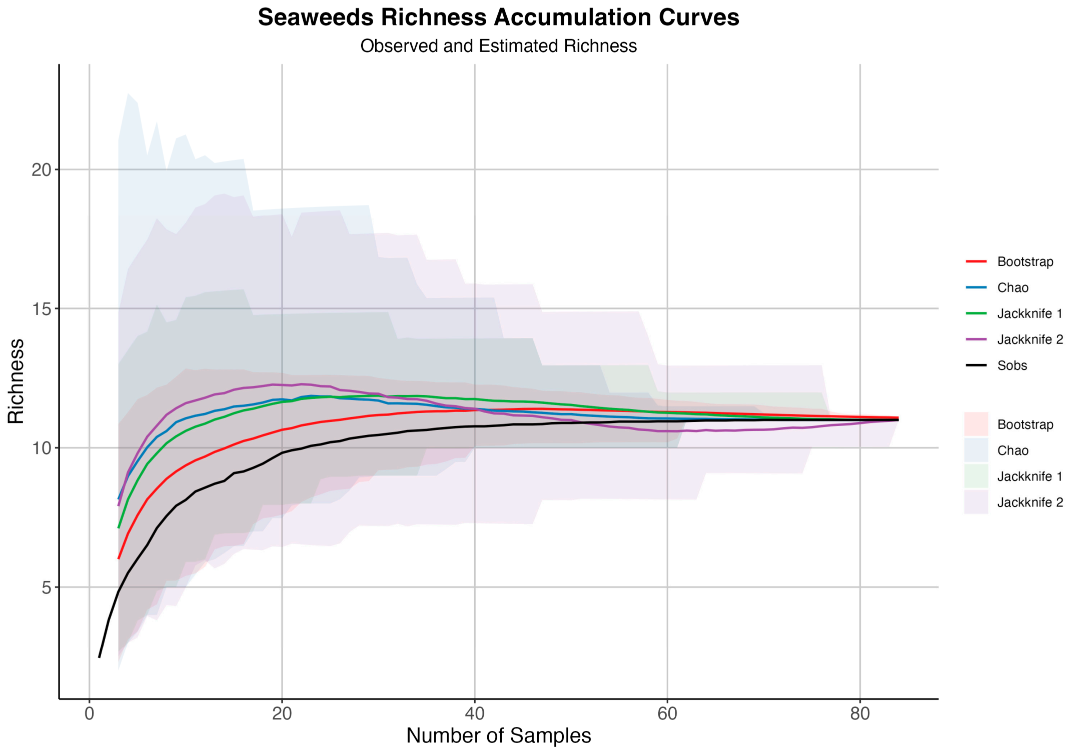


**Figure S2.** Richness accumulation curves for macroalgal genera recorded in the South West Lagoon of New Caledonia. Curves show observed richness (Sobs, black) and non-parametric estimators Bootstrap (red), Chao 2 (blue), first-order Jackknife (Jack 1, green), and second-order Jackknife (Jack 2, purple), plotted as a function of the number of transects (sampling units). All curves tend toward an asymptote, confirming that sampling effort was sufficient to capture the majority of macroalgal generic diversity.

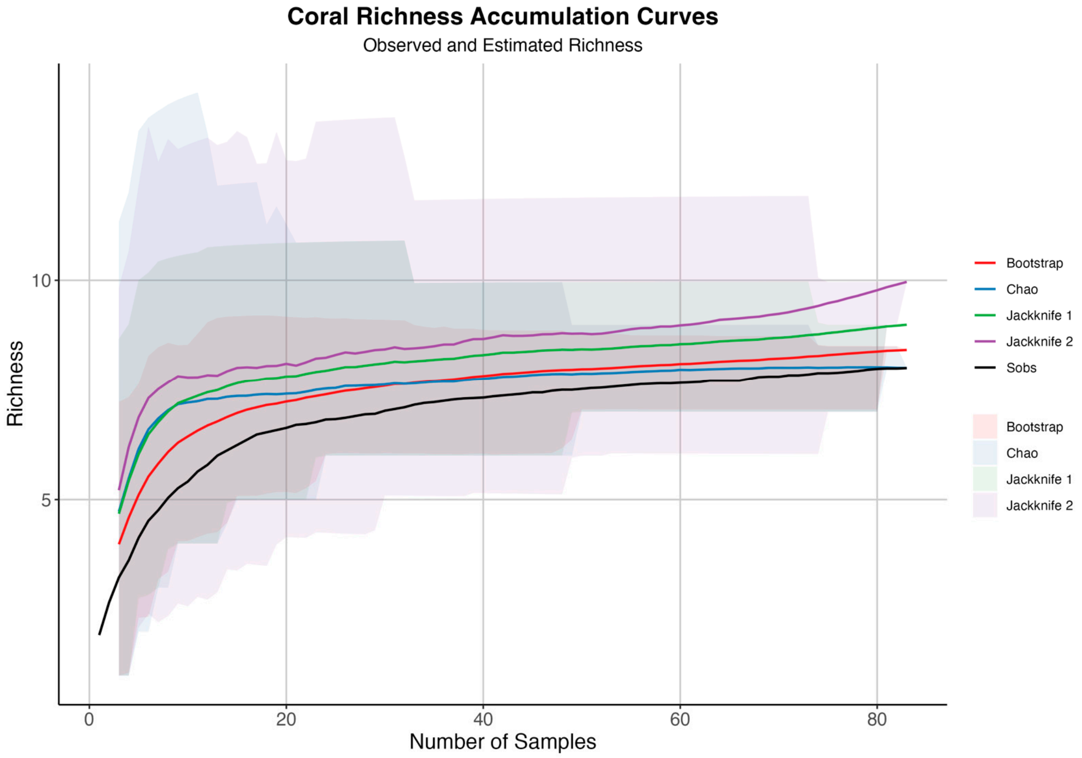


**Figure S3.** Richness accumulation curves for coral genera recorded in the South West Lagoon of New Caledonia. Curves show observed richness (Sobs, black) and non-parametric estimators Bootstrap (red), Chao 2 (blue), first-order Jackknife (Jack 1, green), and second-order Jackknife (Jack 2, purple), plotted as a function of the number of transects (sampling units). The curves approach a plateau, indicating that sampling effort adequately represented coral generic diversity across sampled habitats.

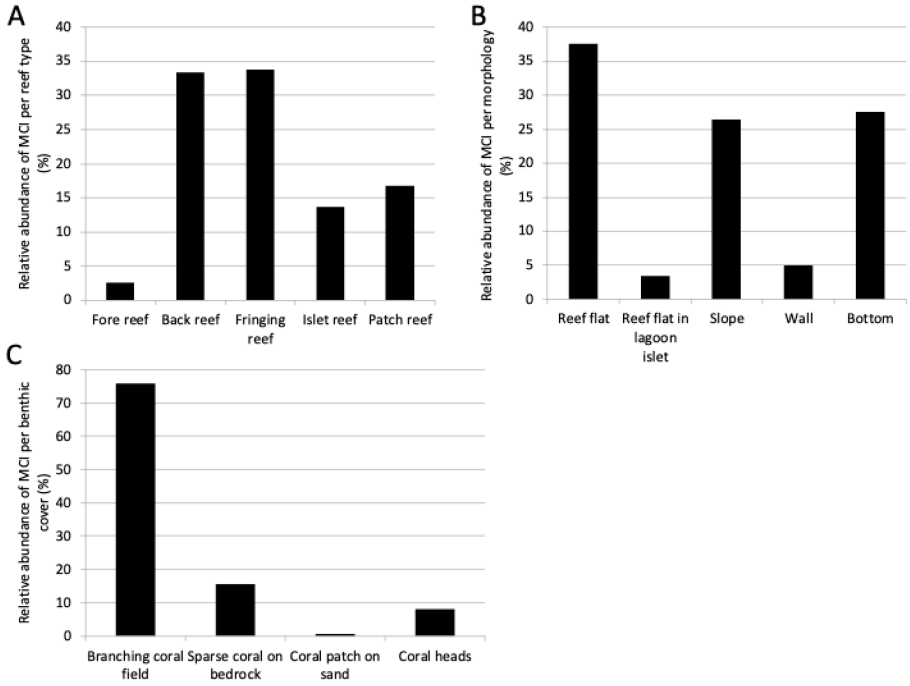


**Figure S4.** Macroalgal–coral interactions relative abundance, in percentage, in the South West lagoon of New Caledonia, per habitat levels (**A**) Reef type, (**B**) Reef zonation, (**C**) Benthic cover.

**Table S1.** Macroalgal-coral interaction (MCI) observed during the preliminary qualitative survey.

| **Coral Genera** | **Macroalga Genera** |
|---|---|
| *Acropora* | *Asparagopsis* |
| *Acropora* | *Caulerpa* |
| *Acropora* | *Ceratodictyon* |
| *Acropora* | *Chlorodesmis* |
| *Acropora* | *Dictyota* |
| *Acropora* | *Galaxaura* |
| *Acropora* | *Halimeda* |
| *Acropora* | *Hypnea* |
| *Acropora* | *Lobophora* |
| *Acropora* | *Padina* |
| *Acropora* | *Sargassum* |
| *Acropora* | *Turbinaria* |
| *Acropora* | Turf |
| *Galaxea* | *Lobophota* |
| *Millepora* | *Dictyota* |
| *Millepora* | *Halimeda* |
| *Millepora* | *Hypnea* |
| *Millepora* | *Lobophora* |
| *Montipora* | *Ceratodictyon* |
| *Montipora* | *Codium* |
| *Montipora* | *Dictyota* |
| *Montipora* | *Halimeda* |
| *Montipora* | *Lobophora* |
| *Montipora* | *Padina* |
| *Montipora* | *Turbinaria* |
| *Montipora* | Turf |
| *Pavona* | *Lobophora* |
| *Pocillopora* | *Dictyota* |
| *Pocillopora* | *Hypnea* |
| *Pocillopora* | *Lobophora* |
| *Pocillopora* | *Turbinaria* |
| *Porites* | *Chlorodesmis* |
| *Porites* | *Galaxaura* |
| *Porites* | *Halimeda* |
| *Porites* | *Hypnea* |
| *Porites* | *Lobophora* |
| *Porites* | *Padina* |
| *Porites* | *Turbinaria* |
| *Porites* | Turf |
| *Seriatopora* | *Chlorodesmis* |
| *Seriatopora* | *Lobophora* |
| *Seriatopora* | Turf |
| *Stylophora* | *Lobophora* |
| *Turbinaria* | *Lobophora* |
